# *Puccinellia maritima*, *Spartina maritime*, and *Spartina patens* Halophytic Grasses: Characterization of Polyphenolic and Chlorophyll Profiles and Evaluation of Their Biological Activities

**DOI:** 10.3390/molecules24203796

**Published:** 2019-10-22

**Authors:** Maria V. Faustino, Maria A. F. Faustino, Helena Silva, Ângela Cunha, Artur M. S. Silva, Diana C. G. A. Pinto

**Affiliations:** 1 QOPNA & LAQV-REQUIMTE, Department of Chemistry, University of Aveiro, Campus de Santiago, 3810-193 Aveiro, Portugal; maria.vf9@ua.pt (M.V.F.); faustino@ua.pt (M.A.F.F.); artur.silva@ua.pt (A.M.S.S.); 2 Department of Biology & CESAM, University of Aveiro, Campus de Santiago, 3810-193 Aveiro, Portugal; hsilva@ua.pt (H.S.); acunha@ua.pt (Â.C.)

**Keywords:** halophytic grasses, UHPLC-MS, phenolic compounds, tricin, chlorophylls, biological activities

## Abstract

Halophytic grasses have been recently targeted as possible sources of nutraceutical and medicinal compounds. Nonetheless, few studies have been conducted on the phytochemistry and biological activities of metabolites produced by these plants. Among these, *Spartina maritima* (Curtis) Fernald, *Spartina patens* (Aiton.) Muhl., and *Puccinellia maritima* (Hudson) Parl. are three halophytic grasses whose chemical composition and bioactivities are unknown. The present work broadens the knowledge on the polyphenolic and chlorophyll composition of these species identifying for the first time hydroxycinnamic acids and their derivatives, flavones, flavonols, lignans, as well as chlorophylls and xantophylls. The extracts were particularly rich in caffeic and ferulic acids as well as in trihydroxymethoxyflavone, apigenin and tricin derivatives. Interestingly, several of the identified compounds are relevant from a medicinal and nutraceutical point of view putting in evidence the potential of these species. Thus, the antioxidant, anti-acetylcholinesterase, antibacterial, and antifungal activities of the polyphenolic extracts were assessed as well as the photophysical properties of the chlorophyll-rich extracts. The results, herein presented for the first time, reinforce the nutritional and the medicinal potential of these halophytic grasses.

## 1. Introduction

The medicinal value of plants has been recognized for thousands of years, and it represents the basis of several traditional medicine systems around the world [1,2,3]. Nowadays, botanical preparations used in folk medicine are being increasingly investigated and continue to provide alternative therapeutic resources [1]. Furthermore, the increasing concern with health and nutrition is bringing a renewed interest on plants as natural sources of nutrients and other compounds associated to health and wellness [4]. Phenolic compounds are in the focus of these investigations due to their diverse chemical structure and wide biological activities, which are valuable in the prevention of chronic or degenerative diseases [3]. In addition, chlorophylls have also gained interest in recent years due to their ability to generate reactive oxygen species (ROS), namely, singlet oxygen (^1^O_2_), which can open new perspectives for application as photosensitizer agents in photodynamic therapy [5]. These molecules are involved in core physiological processes of the plants, but they are also important as food and pharmaceutical industries [6].

Halophytes are plants capable of thriving in saline soils and are gaining interest as food that are involved in the global problem of soil salinization and scarcity of irrigation water. In addition to the high nutritional value, halophytes are also promising sources of bioactive compounds [7,8]. Therefore, halophytic grasses represent an alternative to agriculture in face of the challenges imposed in the near future by global climate change [9], and scientists are starting to correlate their metabolite profile to the saline environment where they can develop [8,10].

*Puccinellia maritima* (Hudson) Parl., *Spartina maritima* (Curtis) Fernald, and *Spartina patens* (Aiton.) Muhl. are three halophytic grasses (Poaceae family) of which the polyphenolic and chlorophyll profiles are, as far as we know, completely unknown. Information on the phytochemistry of the Pucinellia genus is strikingly scarce; with one recent report on the chemical composition of *Puccinellia tenuiflora* [10] and nothing else about the other 110 species of the genus [11]. Similarly, few investigations were performed on the 235 known species of Spartina [11]. Also, there is no information on biological activities of metabolites produced by *P. maritima*, *S. maritime*, or *S. patens.*

The aim of this work is to provide an extensive characterization of the polyphenolic and chlorophyll-rich extracts of *P. maritima*, *S. maritima*, and *S. patens*, as a strategy for the identification of high added-value metabolites. The antioxidant, acetylcholinesterase inhibitor, antibacterial, and antifungal activities of polyphenolic extracts and the photosensitization effect of chlorophyll-rich extracts were explored in the perspective of applications, such as nutraceuticals or photosensitizer agents for photodynamic therapy.

## 2. Results and Discussion

### 2.1. Polyphenolic Rich Extracts UHPLC-DAD-ESI/MS^n^ Analysis

Targeting the identification and quantification of the phenolic constituents of *S. maritima*, *P. maritime*, and *S. patens*, the polyphenolic extracts of these species were analyzed by UHPLC-MS. This analysis resulted in the identification of a total of 65 different compounds (Table 1 and Table S3), 31 in *P. maritima*, 34 in *S. maritima* and 36 in *S. patens*. It was clear that the extracts were particularly rich in hydroxycinnamic acids, such as caffeic and ferulic acids, as well as in flavones, such as trihydroxymethoxyflavone, apigenin and tricin derivatives (Table 1). In addition, one hydroxybenzoic acid (syringic acid derivative) was present in significantly high quantities.

In relation to the total mass of the extract, the proportion corresponding to the compounds identified in *S. maritima*, *S. patens*, and *P. maritima* was not significantly different between species (23.1%, 15.8%, and 13.1%, respectively). This corresponds to an average proportion of 82.6% of non-polyphenolic compounds in the plant extracts. Peaks corresponding to chlorophylls in the extracts can also be observed in the chromatograms (Figure S2) and can contribute to this. Therefore, a profile of chlorophylls was obtained and will be discussed later on. Furthermore, the chromatograms of the extracts corresponding to the three taxa showed that these species are particularly rich in polyphenolic compounds (Figure S2). This is also confirmed by the results of phenolic compounds quantification by Folin–Ciocalteu method (Supplementary Material).

**Figure 1:**
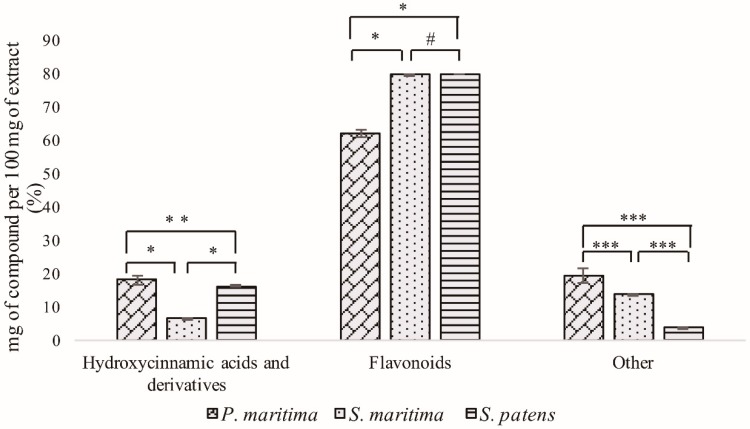
Total amount of each class of compounds in extracts of *Puccinellia maritima*, *Spartina maritime*, and *Spartina patens*. ^*^Statistically different (Tukey’s test) *p* < 0.001; **Statistically different (Tukey’s test) *p* = 0.05; ***Statistically different (Tukey’s test) *p* < 0.05; # Not statistically different (Tukey’s test) *p* = 0.994.

The achieved data revealed that the three studied taxa produce similar amounts of the same chemical families (Figure 1). For instance, *S. maritima* and *S. patens* similar flavonoid content, corresponding to, respectively, 79.7% and 79.8% of the total identified compounds (*p* = 0.994), while the corresponding value in *P. maritima* was 62.2%. Regardless of the similarities of the different plant species in terms of total content of each chemical family, the profile of secondary metabolites varied significantly between species (Table 1). *S. maritima* and *S. patens* produce higher amounts of phenolic compounds, when compared with *P. maritima*. This is coherent with the results of the percentage of identified compounds relative to the total mass of the extract, in which the highest and the lowest values were observed in *S. maritima* and in *P. maritima*, respectively. The Tukey’s test confirmed that the differences were significant (*p* < 0.05), except for the content in flavonoids in *S. maritima* and *S. patens*.

Flavonoids were quantitatively dominant in the polyphenolic extract of *P. maritima* (62.2%), whereas hydroxycinnamic acids and derivatives represented 18.3% of the identified compounds (Table 1 and Figure 1). The major constituent of this extract was a syringic acid derivative **61** with 2.36 ± 0.35 mg/100 mg of extract, followed by a coumaroylferulic acid hexoside derivative **13** (0.62 ± 0.00 mg/100 mg of extract) (Table 1).

The remaining compounds were mostly chlorogenic acids as well as tricin, trihydroxymethoxyflavone, luteolin, and apigenin derivatives. This species showed the highest amount of chlorogenic acids among the three studied taxa (Table 1). Other compounds accounted for 19.4% of the total amount identified (Figure 1).

Flavonoids are also the dominating compounds in polyphenolic extracts of *S. maritima*, representing 79.7% of the total. Hydroxycinnamic acids and derivatives were also present in considerable amounts (6.5%) (Figure 1). Other compounds accounted for 13.7%, and the most represented compound was apigenin-*O*-hexoside **23** (3.14 ± 0.24 mg/100 mg of extract), followed by syringic acid derivative **61** (2.32 ± 0.12 mg/100 mg of extract) and trihydroxymethoxyflavone-*C*-hexoside (isomer II) **25** (1.54 ± 0.14 mg/100 mg of extract) (Table 1). Although the hydroxycinnamic acid derivatives were also represented in *S. maritima* extract, particularly the coumaroylferulic acid hexoside derivative **12** (0.69 ± 0.01 mg/100 mg of extract), the extract was dominated by apigenin, trihydroxymethoxyflavone and tricin derivatives (Table 1).

Similarly, in the polyphenolic extract of *S. patens*, flavonoids represented 79.8% of the total identified compounds. Nonetheless, in this species, the hydroxycinnamic acids were present in higher amounts (16.4%) when compared with *S. maritima* (Figure 1). Other compounds only accounted for 3.8% of the total compounds identified. In this extract, trihydroxymethoxyflavone-*C*-hexoside (isomer I) **19** (0.80 ± 0.05 mg/100 mg of extract) was the most abundant compound, followed by luteolin-8-*C*-hexoside **17** (0.74 ± 0.04 mg/100 mg of extract) and *p*-coumaroylshikimic acid **11** (0.68 ± 0.01 mg/100 mg of extract). The extract was also very abundant on trihydroxymethoxyflavone derivatives as well as on tricin, apigenin, and dihydroxymethoxyflavone derivatives (Table 1).

#### 2.1.1. Hydroxycinnamic Acid Derivatives

Overall, 15 hydroxycinnamic acid derivatives were identified in the extracts of *S. maritima*, *S. patens*, and *P. maritima* (Table 1). These derivatives showed UV spectra consistent with what is described in the literature (Table S3) [4,12,13]. Although cinnamic acids naturally occur in the free form, in the analyzed taxa herein discussed, they appeared mainly linked to quinic acid (compounds **1**, **4**, **5**, and **10**), which is accountable to an important chemical family, the chlorogenic acids. They were also uncovered linked to monosaccharides, which is the case of compounds **3**, **8**, **9**, **12**, and **13** (Table 1). Last, they also appear linked to glycerol (compounds **6**, **7**, and **14**) and to shikimic acid **11** as well as in isoprenyl esters form **2**.

**Figure 2:**
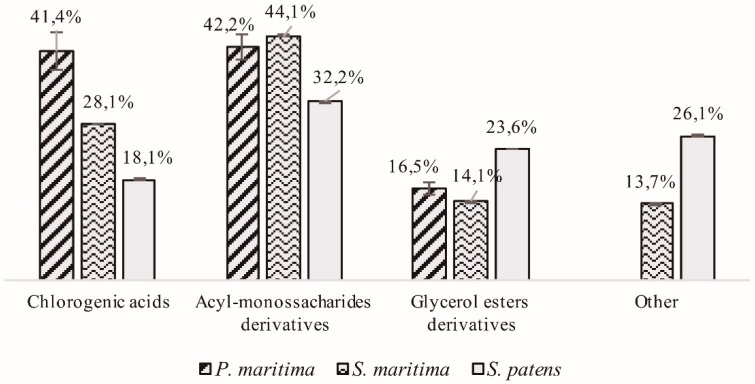
Representative graphic of the hydroxycinnamic acid derivatives in relation to the total hydroxycinnamic acid content identified in *S. maritima*, *P. maritime*, and *S. patens*.

From a quantitative perspective, the extracts of both *P. maritima* and *S. maritima* showed in major quantities of the acyl monosaccharides derivatives, representing 42.2% and 44.2% of the total mass of hydroxycinnamic acids identified, respectively (Figure 2). *P. maritima* has the largest quantity of chlorogenic acids of those species tested, which are the second major type of hydroxycinnamic acids produced by this species (41.4%), followed by glycerol esters derivatives, which accounted for 16.5% (Figure 2). In this species’ extract, no other type of hydroxycinnamic acids was uncovered. The compound from this family found in major quantities was a coumaroylferulic acid hexoside derivative **13** (Table 1). Similarly, in *S. maritima*’s extract, chlorogenic acids was the second major type of hydroxycinnamic acids found (28.1%), followed by glycerol esters derivatives (14.1%). *S. maritima*’s extract also presents other types of hydroxycinnamic acids such as a caffeic acid isoprenyl ester **2** (Table 1). The hydroxycinnamic acid derivative present in major quantities was an isomeric form of the one also reported in high quantity in *P. maritima* (coumaroylferulic acid hexoside derivative **12**) (Table 1). At last, *S. patens* also presented in major quantities acyl monosaccharides derivatives (32.2%). The second major class of hydroxycinnamic acids identified was glycerol esters derivatives (23.6%), followed by other hydroxycinnamic acids (26.1%) and chlorogenic acids (18.1%) (Figure 2). The hydroxycinnamic acid reported in major quantities, in this case, was *p*-coumaroylshikimic acid **11** (Table 1).

The assignment of the different chlorogenic acids (Table 1) was based on the hierarchical keys developed by Clifford and co-workers [14,15]. Consequently, compounds **1** and **4** were, respectively, identified as 3-*O*- and 5-*O*-caffeoylquinic acid, due to pseudomolecular ion [M-H]^−^ at *m*/*z* 353 (Table 1) and their diagnostic ions at *m*/*z* 191 and 179 (Table S3) [4,12,14,16]. As both compounds presented a base peak at *m*/*z* 191, the discrimination between the two was made based on the secondary peaks: the isomer 3-*O*- presented one peak at *m*/*z* 135 [caffeic acid-H-CO_2_]^−^ and the isomer 5-*O*- at *m*/*z* 161 [caffeic acid-H-H_2_O]^−^ (Table S3) [12]. Additionally, the abundances of these peaks are also in agreement with results reported by Clifford and co-workers [14].

Two other members of the chlorogenic acid family, compounds **5** and **10**, were assigned to 3-*O*- and 4-*O*-feruloylquinic acids, respectively, with pseudomolecular ion [M-H]^−^ at *m*/*z* 367. These two feruloylquinic acids are easily distinguishable by their base peak that in the first case corresponded to *m*/*z* 193 and in the second to *m*/*z* 173 (Table S3) [14,17]. 3-*O*-Feruloylquinic acid also showed fragment peaks at *m*/*z* 191 and at *m*/*z* 134, whereas 4-*O*-feruloylquinic acid showed a representative secondary peak at *m*/*z* 193 (Table S3) [14,17].

Other caffeic acid derivatives were also identified. Peak **3** presented a pseudomolecular ion [M-H]^−^ at *m*/*z* 341, releasing MS^2^ fragment ions at *m*/*z* 179 (base peak), corresponding to the deprotonated caffeic acid; the loss of an hexose [M-H-162]^−^, *m*/*z* 161; and a decarboxylation, *m*/*z* 135 (Table S3). These observations, as well as those in the literature information, allowed the identification of compound **3** as caffeic acid hexoside (Table 1) [12,18,19]. Peak **2** showed the same MS^2^ fragment ions. However, the pseudomolecular ion [M-H]^−^ was found at *m*/*z* 247. The base peak was detected at *m*/*z* 179, which correspond to the loss of 68 *m*/*z* (Table S3), and was assigned to isoprenyl moiety [20]. Therefore, this compound was identified as caffeic acid isoprenyl ester (Table 1).

Peaks **6** and **7** presented the same pseudomolecular ion at *m*/*z* 429 (Table 1) and similar MS^2^ mass fragmentation at *m*/*z* 235, 193, 179, 161, and 135. These fragment ions (Table S3) are consistent with a mixed ester of ferulic and caffeic acids, and consequently the two peaks were assigned as isomeric forms of feruloyl-caffeoylglycerol [21,22,23]. Regarding compound **14**, the mass spectra showed peaks indicating the presence of a ferulic acid moiety (*m*/*z* 193) and the pseudomolecular ion, MS^2^ fragment ions, and UV spectrum were in agreement with the literature information for diferuloylglycerol (Table S3) [24,25,26]. Compounds **12** and **13**, with pseudomolecular ions [M-H]^−^ of *m*/*z* 565, were identified as coumaroylferuloyl hexoside derivatives (Table 1), based on the fragment ions observed in MS^3^ (Table S3), which showed a characteristic loss of hexose and ferulic acid moieties [27,28].

Peak **8**, with pseudomolecular ion [M-H]^−^ at *m*/*z* 655 was assigned to a salvianolic acid A hexoside, mainly due to the fragment ions and UV max observed (Table S3). A base peak was detected at *m*/*z* 493, pseudomolecular ion of salvianolic acid A, and ions at *m*/*z* 359 and 179, which are characteristic of this compound (Figure S3) [29,30,31]. Peak **9** ([M-H]^−^ at *m*/*z* 431) showed releasing fragment ions identical to the ones reported by Barros and co-workers (Table S3) [32], also consistent with sinapic acid hexoside (Table 1). Peak **11** showed similar releasing MS^2^ fragments to the ones reported by Said and coworkers [33], including the MS^2^ fragment at *m*/*z* 163 (Table S3), and was therefore assigned as *p*-coumaroylshikimic acid (Table 1) [12,33]. Peak **15** was identified as ferulic acid guaiacylglyceryl derivative (Table 1) because of a pseudomolecular ion [M-H]^−^ at *m*/*z* 613, and a base peak at 569 [M-H-CO_2_]^−^, which corresponds to the loss of 196 *m*/*z* (normally assigned to guaiacylglyceryl moiety) [25,34,35].

Hydroxycinnamic acids are considered the major subgroup of phenolic acids, with a ubiquitous distribution in plants. These compounds have received considerable attention from biomedical research due to their pronounced antioxidant activity [36,37] and protective effects against cardiovascular disorders [36] and cancer [38]. For instance, the chlorogenic acid 5-*O*-caffeoylquinic acid **4**, only present in extracts of *P. maritima* (Table 1), exhibits several biological activities, including antibacterial, antioxidant, hypoglycemic, anticarcinogenic [39,40], and hypolipidemic [41]. Moreover, other promising biological activities have been reported for chlorogenic acids [42], namely, the amelioration of oxidative and inflammatory stress [43] as well as high blood pressure conditions [44] Herein, four chlorogenic acids were unidentified: compounds **5** and **10** were detected in all the investigated taxa and compounds **1** and **4**, corresponding to caffeoylquinic acids, were only found in the extract of *P. maritima* (Table 1).

Caffeic acid derivatives such as caffeic acid isoprenyl ester **2** (only identified in *S. maritima*) and caffeic acid hexoside **3** (only present in *S. patens*) have the ability to improve cell viability [45] and eliminate superoxide radicals [46]. Furthermore, salvianolic acid A hexoside **8**, only detected in *P. maritima*, showed significant protective effect against lipid peroxidation [47]. Moreover, sinapic acid and derivatives are known for antioxidant, anti-inflammatory, antimicrobial, and anticarcinogenic activities [48,49]. In the present study, one sinapic acid hexoside derivative (**9**) was present, and it is only found in the extracts of *P. maritima* (Table 1). *p*-Coumaroylshikimic acid (compound **11**) found in *S. patens*, is considered a promising antioxidant and chemoprotective agent, as well effective in the prevention of cardiovascular disease and cancer [50]. Ferulic acid derivatives are also known for biological activities that include anti-inflammatory, antidiabetic, anticarcinogenic, anti-aging, and neuroprotective effects, among others [51,52,53,54,55]. Herein, two coumaroylferulic acid hexoside derivatives (compounds **12** and **13**) were identified; the former only in *S. maritima* and the later on *P. maritima* and *S. patens* (Table 1). Ferulic acid guaiacylglyceryl derivative **15** was also identified but only in the extract of *S. patens*. Two isomeric forms of feruloyl-caffeoylglycerol were identified: compound **5** in the species and compound **6** only in *P. maritima* (Table 1).

The results confirm that these plant species, especially *P. maritima*, may be regarded as valuable sources of hydroxycinnamic acids derivatives. Due to their interesting biological activities, it is possible to gain perspective on the application of these edible halophytes as nutraceuticals for the prevention of chronic diseases.

#### 2.1.2. Flavonoid Derivatives

**Figure 3:**
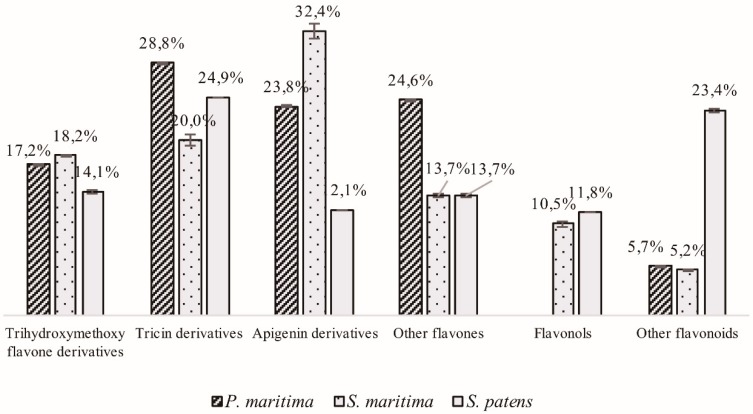
Representative graphic of types flavonoids in relation to total flavonoid content identified in *S. maritima*, *P. maritime*, and *S. patens*.

On the overall, 45 flavonoids were identified in the polyphenolic extracts of the three studied species. In fact, this chemical family dominates the extracts, representing 62.2%, 79.9%, and 79.8% of the total mass of the identified compounds in *P. maritima*, *S. maritime*, and *S. patens*, respectively (Figure 1). Flavones were the most represented subclass, especially tricin and apigenin derivatives, but flavones bearing simultaneously three hydroxyl and one methoxyl groups were also well represented (Figure 3). These flavones have been found in other halophytic species [56,57] and are considered typical of the *Poaceae* family [58].

Tricin derivatives were the most represented flavonoids (28.8%) in *P. maritima* (Figure 3), and tricin *C*-galloyl glucuronide **45** was the predominant derivative found (Table 1).

Other flavones, including luteolin derivatives and quercetagetin trimethyl *O*-sulfate pentoside, represented 24.6% of the identified flavonoids. Apigenin derivatives (23.8%) as well as trihydroxymethoxyflavone derivatives (17.2%) are also well represented (Figure 3). Flavonols were not found in the polyphenolic extract of *P. maritima*.

Apigenin derivatives (32.4%) and, particularly, apigenin-*C*-hexoside **23** (3.14 ± 0.24 mg/100 mg of extract) dominated the extract of *S. maritima* (Table 1). The second most abundant types of flavonoids corresponded to tricin derivatives (20.0%), and trihydroxymethoxyflavone derivatives (18.2%) (Figure 3). Other flavones, like quercetagetin trimethyl *O*-sulfate pentoside and dihydroxymethoxyflavone derivatives, represent 13.7% of the identified compounds (Figure 3). Some flavonols, namely, spinacetin derivatives **51** and **52**, kaempferol galloyl hexoside **55**, and methyl catechin acetate glucuronide **60**, were also identified in the extract of *S. maritima* (Table 1).

In the polyphenolic extract of *S. patens*, tricin derivatives were identified as the most represented flavonoids (24.9%) (Figure 3). Other flavonoids, representing a total of 23.4%, include homoisoflavonoids (hydroxydimethoxydimethyl homoisoflavone and methylginestein derivatives), as well as flavanones (erioctioyl di-*O*-hexoside). Trihydroxymethoxy-flavone derivatives were the third most represented type of flavonoids (14.1%) (Figure 3). In comparison with the other species, apigenin derivatives were poorly represented in the extract of *S. patens*. Although *S. maritima* and *S. patens* are included in the same genus, a clear differentiation among the polyphenolic profile of the two species was observed. All the identified flavonoids presented a typical UV–Vis spectrum with two absorbance bands: band A in 350–385 nm for flavonols and 310–350 nm for flavones, and band B in 250–290 in all types of flavonoids [12].

● Tricin derivatives

As previously stated, tricin derivatives are one of the major types of flavonoids found in this study. In all tricin derivatives, an MS^2^ ion at *m*/*z* 329 [tricin-H]^−^ can be detected, supporting the final identification. In the case of the UV spectra, these compounds showed band A at 300–400 nm, corresponding to the cinnamoyl system of ring-B, and band B at 240–280 nm, corresponding to the benzoyl system of ring-A [59]. These flavone derivatives are common in Poaceae, such as wheat, rice and barley [60]. Additionally, this flavone normally occurs as lignan and lignan–glycoside derivatives [60].

Compound **48**, with pseudomolecular ion [M-H]^−^ at *m*/*z* 329 [tricin-H]^−^, revealed MS^2^ diagnostic fragments of tricin at *m*/*z* 314 (base peak), due to loss of CH_3_ and at *m*/*z* 299 due to loss of two CH_3_ groups, and was therefore assigned to tricin. This agrees with the typical mass fragmentation of tricin, described by several authors [61,62,63]. Compounds **31** ([M-H]^−^ 491 *m*/*z*), **38** ([M-H]^−^ 373), **40** ([M-H]^−^ 409) and **43** ([M-H]^−^ 403), all showed a MS^2^ base peak at *m*/*z* 329 [tricin-H]^−^. In the first one, a loss of 162 *m*/*z* is compatible with an *O*-hexose and therefore, the compound was assigned as tricin-*O*-hexoside. As the UV spectrum showed maxima at 269 nm and 351 nm (Table S3) the isomer could be deduced as 7-*O*-hexoside (Figure S4), meanwhile the 5-*O*- present UV maxima at 297 and 381, according to Li et al. [60]. Peak **38** showed a loss of 44 *m*/*z* correspondent to an acetyl group [64]. This compound was identified as 3-*O*-acetyltricin since this isomer is commonly found in Poaceae [60]. In compound **40**, a loss of 80 *m*/*z* equivalent to a sulfate group was detected [65], assigning this compound to tricin sulfate. Peak **43** was identified as tricin glyceryl since it showed a loss of 74 *m*/*z* equivalent to dehydrated glycerol [66]. Compounds **34** and **36** showed the same pseudomolecular ion [M-H]^−^ at *m*/*z* 687 and similar MS^2^ fragment ions at *m*/*z* 525 (base peak) [M-H-hexose] and 329 [Tricin-H]^−^ with loss of 196 *m*/*z*, typical of a guaiacylglyceryl group [25,60,67], and were identified as flavonolignans tricin guaiacylglycyl hexoside isomers I and II (Table 1) [25,60]. Additionally, compound **42** presented a base peak of 329 [tricin-H]^−^, which correspond to a loss of 276 *m*/*z*. This could be due to the loss of sulfated guaiacylglyceryl (80 + 196) and, therefore, was tentatively assigned as tricin guaiacylglycerylsulfate, which was already reported in Poaceae [60]. Compound **50** showed the base peak at *m*/*z* 329 with loss of 196 *m*/*z* equivalent to a guaiacylglyceryl moiety. Therefore, the compound was tentatively assigned as tricin-4-*O*-guaiacylglyceryl (Figure S4) since this is the most common position in which guaiacylglyceryl group is found [25,60].

Another tricin lignan with a pseudomolecular ion [M-H]^−^ at *m*/*z* 853, was identified (compound **53**). This compound was tentatively assigned as tricin secoisolariciresinol coumaroyl. The first sign of the presence of this lignan was the MS^2^ fragment ion at 805 *m*/*z* correspondent to the loss of 48 *m*/*z*. This loss is normally found in lignans with a hydroxymethoxy group, losing easily the formaldehyde in negative ion mode, which is the case of secoisolariciresinol. The base peak at *m*/*z* 493, with the loss of 360 *m*/*z* is also in agreement with the presence of secoisolariciresinol [68]. Another peak supporting the identification occurred at *m*/*z* 329 [tricin-H]^−^, corresponding to [M-H-secoisolariciresinol-164]^−^, which is normally equivalent to the loss of a coumaroyl moiety [69]. Peaks **45** and **47** showed pseudomolecular ions [M-H]^−^ of 691 and 643, respectively. Their MS^2^ fragment ions showed, in the first case, loss of a glucuronic acid [M-H-194]^−^, and in the second, of an *O*-hexoside [M-H-162]^−^. In these compounds, peaks correspondent to the loss of a methyl galloyl moiety [M-H-168]^−^ or a galloyl moiety [M-H-152]^−^, was also detected. Therefore, the compounds were assigned as tricin *C*-methylgalloyl glucuronide and tricin-*C*-galloyl-*O*-hexoside, respectively [70,71].

Several biological activities are attributed to free tricin but also to tricin-glycosides, tricin-lignans, and tricin-lignans-glucosides [60]. Tricin **48**, detected in the three studied species, and tricin-guaiacylglyceryl **50** (detected in *S. maritima* and *P. maritima*) have antioxidant and anti-inflammatory activities in carrageenan-induced paw edema experiments [72]. Additionally, several activities such as anti-allergy [73], antiplatelet aggregation [74], antitumor [75], and cardioprotective activities [76] have been reported for tricin lignans. The cluster corresponding to flavone and its derivatives was characterized by a wide diversity among all studied species, and the above mentioned biomedical applications contribute to increase these species value.

● Apigenin derivatives

Apigenin and their derivatives showed a strong band A at 320–400 nm and band B at 290–320 nm [77], which is in agreement with the data found in the extracts herein investigated. Additionally, all the apigenin derivatives presented the aglycone peak at *m*/*z* 269 or the respective [aglycone+41]^−^ and [aglycone+71]^−^ in the cases of monoglycosides derivatives or [aglycone+113]^−^ and [aglycone+83]^−^ in diglycosides [78].

Peaks **23**, **32**, and **35** exhibited the same pseudomolecular ion [M-H]^−^ at *m*/*z* 431 and diagnostic MS^2^ fragment ions. In the first case, an MS^2^ ion at 269 *m*/*z* [M-H-162]^−^ equivalent to an *O*-hexose was found [78], and therefore the compound was identified as apigenin-*O*-hexoside. In compounds **32** and **35**, an MS^2^ fragment ions at *m*/*z* 341 [M-H-90]^−^, corresponding to [aglycone+71]^−^ and at *m*/*z* 311 (base peak) [M-H-120]^−^ equivalent to [aglycone+41]^−^, were observed. This fragment pattern is typical of *C*-hexoside derivatives assigning these compounds to apigenin-*C*-hexosides. According to Pikulski and Brodbelt [79], the differentiation between the two *C*-hexoside isomers relies on the abundance of the peak at *m*/*z* 341. This is more abundant in the case of the 6-*C*-hexoside when compared to 8-*C*-hexoside. Therefore, compound **32** was tentatively assigned as apigenin-6-*C*-hexosides and peak **35** as apigenin-8-*C*-hexosides (Figure S4). Compounds **20** and **22** were, respectively, identified as apigenin-di-*C*-hexoside and apigenin-di-*C*-pentoside. In both MS^2^ fragment spectra, the ions correspondent to [aglycone+113]^−^ and [aglycone+83]^−^ were present. In the first case, a peak at *m*/*z* 473 due to loss of 120 *m*/*z*, typical of *C*-hexose, was observed [80]. Regarding compound **22**, the peaks equivalent to [M-H-60]^−^ and [M-H-90]^−^, typical of *C*-pentose, were observed [80,81]. Peak **18** presented a pseudomolecular ion [M-H]^−^ at *m*/*z* 563 and MS^2^ diagnostic fragment ions at *m*/*z* 473 [M-H-90]^−^, *m*/*z* 443 [M-H-120]^−^, *m*/*z* 383 [M-H-120-60]^−^, and *m*/*z* 353 [M-H-120-90]^−^ which suggest a glucosyl and a penstosyl group in position 6 and 8, typical of *C*-glycosylflavones [12,80]. An ion at *m*/*z* 545, although with low intensity, is consistent with a loss of [M-H-60]^−^ which is in agreement with a *C*-pentosyl cleavage and the fragment ion [M-H-120]^−^, consistent with a *C*-hexosyl cleavage, allowed us to propose the structure for this compound as apigenin-8-*C*-pentose-6-*C*-hexose. However, these results are also in agreement with the structure of apigenin-8-*C*-hexose-6-*C*-pentose [82]. Peak **29**, with pseudomolecular ion [M-H]^−^ at *m*/*z* 769, was tentatively identified as apigenin-*C*-hexoside-*O*-caffeoylglucoronide. This identification was based on diagnostic ions in the MS^2^ spectrum, such as the ion at *m*/*z* 593 [M-H-176]^−^, which can be attributed to the loss of glucuronide moiety [83]. The ion at *m*/*z* 413 (base peak) [M-H-176-180]^−^ accounts for the loss of a caffeoyl moiety [84], and the ions at *m*/*z* 323 [M-H-caffeoylglucuronide-90]^−^ and *m*/*z* 293 [M-H-caffeoylglucuronide-120]^−^ indicate the presence of a *C*-hexose [80]. Compound **30** exhibited a base peak at *m*/*z* 537 and MS^2^ fragment at *m*/*z* 375, which indicates that the product ion underwent a retrocyclization fragmentation [85].

Additionally, the values of UV absorption (269, 332 nm) in the UV spectrum are typical of a biflavonoid [86]. Therefore, the compound was assigned as biapigenin (amentoflavone).

Biological activities are well known in apigenin and its derivatives. Apigenin-7-*O*-glucoside is a potent anticandidal agent and presents cytotoxic activity against colon cancer cells [87]. Other apigenin derivatives show antibacterial [88] and antitumoural activity, namely, against colorectal adenocarcinoma (HT-29) and leucocythemia (HL-60) cell lines [89]. The presence of high amounts of these compounds in the extracts of the studied halophytes emphasizes the potential of the studied species as sources on natural pharmaceuticals.

● Other flavones

Several trihydroxymethoxyflavone derivatives were identified in the extracts of *S. maritima*, *S. patens*, and *P. maritima*. The UV maximum was, in all cases, ~270 nm (band B) and 350 nm (band A), matching the values described in literature for this type of derivatives [90,91]. In all cases, the peak correspondent to the aglycone was observed at *m*/*z* 299, suggesting that the presence of the diosmetin or chrysoerisol derivatives, such as trihydroxymethoxyflavones, is widespread in nature [90,91,92].

The same pseudomolecular ion at *m*/*z* 461 appears for peaks **19**, **25**, **27**, **33**, and **37** and diagnostic MS^2^ fragment ions at *m*/*z* 371 [M-H-90]^−^ and at *m*/*z* 341 (base peak) [M-H-120]^−^, which indicates the presence of a *C*-hexosyl group. The MS^3^ of 341 showed, in all cases, the the aglycone presence at *m*/*z* 299. This leads to the identification of these compounds as trihydroxymethoxyflavone-*C*-hexoside I, II, III, IV, and V, which is also in agreement with the literature [90,91,92]. Peak **58** was identified as trihydroxymethoxyflavone-*O*-hexoside-*O*-malloyl. This identification was based on MS^2^ diagnostic peaks at *m*/*z* 415, due to loss of an *O*-hexose [M-H-162]^−^, and at *m*/*z* 299, due to loss of 116 *m*/*z* [93].

Peak **59** exhibited a pseudomolecular ion at *m*/*z* 649 and MS^2^ fragment ions at *m*/*z* 487 [M-H-162]^−^ due to loss of an *O*-hexose, *m*/*z* 413 [M-H-162-74]^−^, which can correspond to glycerol [66], and at *m*/*z* 299 (base peak) [M-H-162-74-114]^−^ equivalent to the loss of hydroxymethoxypentanoic acid [33]. Therefore, the compound was assigned to trihydroxymethoxy flavone *O*-glycosylhexoside-*O*-5-hydroxy-4-mehoxypentanoic acid.

Apart from the three major flavone derivatives herein uncovered, two luteolin derivatives corresponding to peaks **17** and **26** were also detected. Both compounds showed a pseudomolecular ion [M-H]^−^ at 447 *m*/*z* and characteristic MS^2^ fragment ions at *m*/*z* 357 [M-H-90]^−^ and *m*/*z* 327 (base peak) [M-H-120]^−^ which indicates the presence of a *C*-glucosyl group and its typical of luteolin-*C*-hexoside [79]. Poaceae predominantly synthesize flavone *C*-glycosides forms, mainly 6-*C* and/or 8-*C*-glycosidic conjugates [94]. Therefore, these two isomers are likely to correspond to the luteolin 6-*C* and the 8-*C*-hexoside derivatives.

Trihydroxymethoxyflavones have been associated to different biological activities. For instance, 3,4′,5-trihydroxymethoxyflavone has a broad-spectrum antibacterial activity [95]. Although these types of flavones were found in all studied extracts, they were particularly abundant in *S. maritima* (compound **25** corresponded to 1.54 mg/100 mg of extract), and several of them were only detected in this species (Table 1). Taking this into consideration, it would be worth exploring the role of these compounds in species-specific physiological traits or mechanisms of adaptation to saline stress.

At last it should be emphasized that a vast array of biological activities has been associated to luteolin and/or to its derivatives. For example, antioxidant activity were demonstrated in DPPH^•^, ABTS^•+^ and FRAP assays [96], as well as anticarcinogenic [97] and anti-inflammatory activity [98]. Two luteolin derivatives were uncovered during this study: compound **17** was present in significant quantities (0.60-0.74 mg/100 mg of extract) in the 3 plant species, and compound **26,** only identified in *P. maritima* (Table 1).

● Flavonol derivatives

Only a few flavonols and four aglycone types—quercetagetin, kaempferol, spinacetin, and gomphrenol—were identified in the extracts of plants of the three studied species.

Two spinacetin derivatives were uncovered, only in *Spartina* species, and their identification was based on UV absorption maxima at 270 and 365 nm (Table S3), which are equivalent to the ones reported in literature for these compounds [99]. Peaks, **51** and **52**, showed pseudomolecular ion [M-H]^−^ at *m*/*z* 541 and 561, respectively. In both MS^2^ mass spectra, a fragment ion at *m*/*z* 345 was observed, which corresponds to the aglycone spinacetin (Figure S4) [98]. In the former, a loss of 196 *m*/*z* equivalent to guaiacylglyceryl moiety [26,60,67] was observed, and therefore the compound was tentatively assigned to spinacetin guaiacylglyceryl. The second spinacetin derivative showed a loss of 224 *m*/*z*, which can correspond to a sinapic acid moiety [100], and therefore the compound was tentatively identified as spinacetin sinapoyl.

One kaempferol derivative (peak **55**) was also identified with pseudomolecular ion [M-H]^−^ of 599 *m*/*z*. The UV absorption maxima at 262 nm and 362 nm (Table S3), typical of kaempferol, were used for the identification [92]. Additionally, in the MS^2^ mass spectrum, a fragment ion corresponding to the aglycone kaempferol was detected, as well as the loss of a galloyl moiety [71,92,101]. Another fragment ion at 447 *m*/*z* (base peak) correspondent to the loss of an hexose [M-H-162]^−^ was observed. Therefore, the compound was assigned to kaempferol galloyl hexoside (Table 1).

Furthermore, two trihydroxymethylenedioxyflavone derivatives were identified. Peak **16** presented a pseudomolecular ion [M-H]^^−^^ at *m*/*z* 653 and MS^2^ fragment ions at *m*/*z* 445 (base peak), due to loss of hydroxyferulic acid [M-H-209]^^−^^ and at *m*/*z* 313, which corresponds to gomphrenol [80,102]. Additionally, the UV absorption maxima are also in agreement with the literature data [102]. Therefore, the compound was assigned as gomphrenol-*O*-pentosyl-*O*-hydroxyferuloyl (Table 1). Peak **41** revealed a pseudomolecular ion [M-H]^^−^^ of 629 *m*/*z* and MS^2^ fragment ions at *m*/*z* 467 (base peak), corresponding to the loss of an *O*-hexose [M-H-162]^^−^^. The MS^3^ fragment ions showed as base peak *m*/*z* 313 [gomphrenol-H]^^−^^ by the loss of 154 *m*/*z*, which according to the literature, can be assigned to dihydrogalloyl [80].

Finally, compound **49** was identified as quercetagetin trimethyl *O*-sulfate pentoside (Figure S4). It showed MS^2^ fragment ions at *m*/*z* 439 (base peak) due to loss of a pentosyl moiety [M-H-132]^^−^^ and at *m*/*z* 359 [M-H-pentose-80]^^−^^, corresponding to the loss of a sulfate group and to the quercetagetin trimethyl aglycone [103]. This type of compounds is common in Poaceae, which ultimately supports the identification [103].

Kaempferol and some of its derivatives are documented in literature as having antimicrobial and antioxidant activities [104]. More recently, the ability to inhibit the growth of HT-29 human colon cancer cells and anti-inflammatory property were also demonstrated [105,106]. In the case of spinacetin, the anti-inflammatory activity is related to the inhibition of histamine release and the production of inflammatory mediators, such as leukotriene C4 (LTC4) and interleukin-6 (IL-6), in IgE/Ag stimulated BMMCs at concentrations of 1, 2, and 5 μM [107]. The flavanols occurrence is more prevalent in *S. maritima* and *S. patens*, which can configure a genus-specific trait.

● Other polyphenolic derivatives

In addition to the compounds previously discussed, two homoisoflavonoids were identified (peak **28** and **44**) with pseudomolecular ions [M-H]^−^ at 815 and 507 *m*/*z*, respectively. These compounds were identified as hydroxydimethoxydimethyl homoisoflavone derivatives. The UV absorption maxima (Table S3) were in accordance with what is reported in literature [108]. Additionally, the MS^3^ fragments at 339 *m*/*z* [aglycone-H]^−^ and at 311 *m*/*z* are typical of homoisoflavones [108]. The base peak in the MS^2^ spectrum at 507 *m*/*z* was equivalent to the loss of 308 *m*/*z* which can be attributed to coumaroylhexoside [146+162-H]^−^, whereas the peak at *m*/*z* 339 corresponded to the loss of 168 *m*/*z* [methylgalloyl-H]^−^ [71]. Therefore, compound **28** was tentatively identified as hydroxydimethoxydimethyl homoisoflavone *O*-coumaroyl-hexoside-*C*-methylgalloyl. The analysis of peak **44** showed a loss of 168 *m*/*z*, again corresponding to the loss of methylgalloyl. Therefore, the compound was assigned to hydroxydimethoxydimethyl homoisoflavone *C*-methylgalloyl [71]. One flavanone (compound **39**) with a pseudomolecular ion of 611 *m*/*z* was also detected and assigned to erioctioyl derivative. In the UV spectrum, absorption maxima at 283 and 327 nm are in accordance with the literature. Additionally, the MS^3^ mass spectra showed a base peak at *m*/*z* 287 [aglycone-H]^−^ characteristic of erioctioyl [109]. Furthermore, two losses of 162 *m*/*z* equivalent to *O*-hexose were observed, and the compound was therefore identified as erioctioyl di-*O*-hexoside. An isoflavanone (compound **56**) with a pseudomolecular ion at 599 *m*/*z* was identified as a methylgnistein derivative, mainly due to the UV spectrum and aglycone mass, which are coincident with previous reported values [102]. It showed MS^2^ peaks at *m*/*z* 419 [M-H-caffeoyl]^−^ and at *m*/*z* 283 [methylgnistein-H]^−^ (loss of 136 *m*/*z*). So the compound was tentatively assigned as methylgnistein caffeoyl derivative.

A catechin derivative (peak **60**), with pseudomolecular ion at 540 *m*/*z* was detected. The UV absorption maxima at 260 and 360–380 nm are in agreement with the literature [110]. The MS^2^ spectrum showed a characteristic peak of *O*-methyl catechin at *m*/*z* 304 [26]; a MS^2^ peak at *m*/*z* 480 (loss of 60 *m*/*z*) is characteristic of an acetyl moiety [111]. Additionally, the peak at 304 *m*/*z* corresponds to the loss of 176 *m*/*z* equivalent to glucuronide moiety [83]. Therefore, the compound was tentatively identified as *O*-methylcatechin acetate glucuronide (Table 1).

#### 2.1.3. Other Compounds

In the whole of the extracts of *P. maritima*, *S. maritime*, and *S. patens*, five more compounds were identified. Compound **61** ([M-H]^−^ at *m*/*z* 241) showed a base peak at *m*/*z* 197 [syringic acid-H]^−^ and was assigned to a syringic acid derivatives [112]. The tetrahydrofuranolignan lariciresinol was deduced based on previously reported mass spectra with the ion correspondent to the aglycone at *m*/*z* 359 and the typical fragment at *m*/*z* 329, due to loss of two CH_3_ groups [68]. Additionally, the losses of two hexoses were also observed, being therefore identified as lariciresinol dihexoside. The identification of other compounds was also based on the pseudomolecular ion and fragments. For some of these compounds interesting biological activities are reported in literature. Lariciresinol inhibits ROS generation in a dose-dependent manner without exhibiting any cytotoxicity [113] and costunolide presents, anti-inflammatory, antitumoral, and antimicrobial activities, among other types of biological activity [114]. The presence of these compounds highlights the diversity of the polyphenolic compounds associated with *P. maritima*, *S. maritime*, and *S. patens*.

### 2.2. Chlorophyll-rich Extracts UHPLC-DAD-ESI/MSn Analysis

Aiming at extracting the highest possible amounts of chlorophylls three extraction approaches techniques were used: microwave-assisted extraction, ultrasound-assisted extraction, and room-temperature extraction under magnetic stirring. To identify the individual pigments present in the chlorophyll-rich extracts, ultrahigh performance liquid chromatography–mass spectrometry (UHPLC-MS) analysis was performed. The identification of the compounds was performed based on pseudomolecular ion [M+H]^+^, retention time and UV–Vis spectra (Table 2).

The UHPLC-MS analysis leads to the identification of the major peaks in each individual chromatogram. In all extracts xanthophylls (lutein and/or zeaxanthin) were identified in addition to chlorophylls. In the case of *P. maritima*, the room temperature stirring extract (*Pm*ST) showed a total absence of peaks in significant quantities, possibly due to the low chlorophylls content in this extract. Nonetheless, in microwave assisted extract (*Pm*MW) and ultrasound assisted extract (*Pm*US) the same pigments were found although with different abundances. In *Pm*MW, the two peaks representing the highest percentage of compounds were assigned to xanthophylls (Table 2). Due to their pseudomolecular ion [M+H]^+^ (569 *m*/*z*), it was possible to deduce that lutein and zeaxanthin were present [115]. Additionally, the first two xanthophylls to elute were identified as lutein and zeaxanthin, respectively, since this elution order has been extensively described in positive ion mode [115,116,117].

Chlorophyll *b* was also identified with pseudomolecular ion [M+H]^+^ at *m*/*z* 885 and with a relatively high peak area percentage. At last, two pheophytin *a* diastereomers were identified based on pseudomolecular ion [M+H]^+^ (*m*/*z* 871) [118,119]. The peak corresponding to pheophytin a I was the one representing the largest percentage in all extracts of *S. maritima* (Table 2). In these extracts, carotene was also identified based on pseudomolecular ion [M+H]^+^ (*m*/*z* 537) and UV–Vis spectra [115,120]. Carotene was the second most abundant peak in *Sm*MW. In addition, two diastereomers of chlorophyll *b* were also identified in *S. maritima*’s chlorophyll-rich extracts (Table 2).

At last, regarding the extracts of *S. patens*, *Sp*MW presented a higher percentage of pheophytin *a* diastereomers (32.4%), followed by lutein (27.4%) and the diastereomers of chlorophyll *b* (15.0%); whereas, in *Sp*ST, carotene corresponded to the largest percentage (36.8%), followed by a diastereomers of chlorophyll *b* (35.4%) and lutein (15.5%). Finally, in *Sp*US extract, a diastereomer of pheophytin *a* was the most represented compound, representing 91.8% of peak area (Table 2).

Pheophytin *a* has already been reported for its photosensitizer properties, with significant inhibition of the survival of HUH-7 cells (hepatocyte-derived cellular carcinoma cell line) [121]. This chlorophyll derivative exhibits basic structural skeleton of chlorin and absorbs strongly in red region [120], which represents an advantage in terms of a potential application of this compound as photosensitizer. Within the xanthophyll pool, lutein has scavenging properties for superoxide radicals, hydroxyl radicals and inhibits in vitro lipid peroxidation [122]. Similar properties have been reported for zeaxanthin and carotene [123,124].

### 2.3. Evaluation of Antioxidant Activity of the Polyphenolic-Rich Extracts

The antioxidant activity of polyphenol-rich extracts of *S. maritima*, *S. patens*, and *P. maritima* was for the first time screened by DPPH, ABTS, and FRAP assays. The DPPH and ABSTS assays were used to evaluate the radical scavenging potential. The results are summarized in Table S7. The IC_50_ values relative to ABTS^+•^ were 81.09 ± 6.24, 67.56 ± 12.82, and 37.13 ± 1.44 µg/mL in *P. maritima*, *S. maritime*, and *S. patens*, respectively. The DPPH scavenging activity of each extract produced IC_50_ values of 373.45 ± 28.63 for *P. maritima*, 317.46 ± 35.68 for *S. maritime*, and 207.63 ± 10.50 for *S. patens*. The results of the DPPH assay showed a similar trend to those of the ABTS^•+^ assay but within a higher range, as observed in other studies [4,125]. The values of IC_50_ corresponding to the extract of the plants were significantly higher than the value obtained with trolox, included as reference compound (Table S7). None of the extracts displayed activity when tested by the ferric reducing antioxidant power (FRAP) assay (values above 500 µg/mL in all cases). Nonetheless, the iron reducing capacity does not necessarily reflect antioxidant activity, as it has been suggested by Wojdyło et al. [125].

The significant lower IC_50_ values presented by extracts of *S. patens* in comparison with extracts of the other halophytes can be related with the content in flavonoids and hydroxycinnamic acids. The activity of flavonoids is directly associated with the structure and substitution pattern of hydroxyl groups. The configuration 3′,4′-*ortho*-dihydroxy is considered essential for the effective radical scavenging activity. Furthermore, the presence of 3-OH or 3- and 5-OH groups is also considered beneficial for the antioxidant activity of flavonoids. The presence of the C2–C3 double bond configured with a 4-keto arrangement is also known to increasing the radical-scavenging activity [125]. *S. patens* produces several flavones with 3-OH and 5-OH substituents, some of which are exclusively produced by this species and in significant quantities (Table 1). The antioxidant effect of hydroxycinnamic acids has also been reported [36,126], and is considered to be associated with structural features like (i) the substituents on the aromatic ring, (ii) the number and position of the hydroxyl groups in relation to the carboxyl group, (iii) position and nature of esterification. It was verified that molecules with *ortho* or *para* dihydroxy groups on the phenyl, as well as molecules with 4-hydroxy groups or with methoxy groups, possess higher antioxidant activity. Additionally, esterification to glycosides maintains or increases the antioxidant properties and esterification in the primary hydroxyl group of the glycoside improve radical scavenging activity [36,126]. Most of the hydroxycinnamic acids identified in the studied extracts have one or more of the above-discussed traits. As an example, the two identified *O*-caffeoylquinic acids (**1** and **4**) reported only in *P. maritima*, present the 4-hydroxy group while caffeic acid hexoside **3**, reported only in *S. patens*, shares the later feature but presents also a glucoside group that could enhance the antioxidant activity. In addition, *p*-coumaroylshikimic acid **11**, diferuloylglycerol **14** and ferulic acid guaiacylglyceryl derivative **15**, which were only found in extracts of *S. patens* (Table 1), also exhibit the features that enhanced the antioxidant activity.

### 2.4. Inhibition of Acetylcholinesterase by the Polyphenolic Rich Extracts

An adaptation of the Ellman’s method was used to measure the inhibition of acetylcholinesterase by the polyphenolic extracts of *S. maritima*, *S. patens*, and *P. maritima* [127]. The extracts caused modest acetylcholinesterase inhibitions (Table S7) and only at the highest tested concentration (100 µg/mL). Because of the very high concentrations expected to be required to produce stronger inhibitions, it was not possible to determine the IC_50_ value.

### 2.5. Photophysical Properties of the Chlorophyll-rich Extracts

For the evaluation of the photophysical properties of the chlorophyll-rich extract, the extract with the highest amount of chlorophylls from each plant species were selected. The selection was based on three parameters: (i) extraction yield, (ii) total chlorophyll content, and (iii) UHPLC-MS analysis. The methodology used to achieve the total chlorophyll content as well as the results from the first two parameters are available on the supplementary material. The extracts selected were microwave assisted extracts of *P. maritima* (*Pm*MW) and *S. patens* (*Sp*MW) and room temperature magnetic stirring from *S. maritima* (*Sm*ST).

#### 2.5.1. Optical Properties and Photostability

All the chlorophyll-rich extracts (*Pm*MW, *Sm*ST, and *Sp*MW) present a similar UV–Vis spectra dominated by one strong absorption band c.a. 400 nm (Soret band) and a last intense absorption band in the red region, c.a. 660 nm, typical of chlorophylls and their derivatives.

The photodegradation studies of the chlorophyll-rich extracts were assessed under red light (10 mW cm^−2^) and white light (50 mW cm^−2^). No photodegradation of *Pm*MW under red light (630 ± 20 nm) was observed since the absorption at 0 min was not significantly different from that collected at 75 min of irradiation. Nonetheless, *Sm*ST and *Sp*MW extracts, under the same irradiation conditions, showed a decrease of 25% and 18% at the Soret absorption after 75 min of light irradiation, respectively. The Tukey’s test confirmed that differences observed are statistically significant only between *Pm*MW and *Sm*ST (*p* = 0.040). Under white light (400–800 nm) irradiation at irradiance of 50 mW cm^−2^, similar profiles were obtained. Under white light conditions, the chlorophyll-rich extract photodegradation increase slightly but keep a similar profile among the extracts, showing after 30 min of irradiation 13% of photodegradation for *Pm*MW, 11% for *Sp*MW and 30% for *Sm*ST. However, differences are not statistically significant (One Way ANOVA, *p* = 0.485). Thus, *Pm*MW showed the best photostability under white and red light. This fact is probably related with the presence on *Pm*MW chlorophyll-rich extract of an important content of xanthophylls (lutein and zeaxanthin) and carotenes with scavenging properties which avoid the degradation of chlorophylls by the formed ROS [122].

#### 2.5.2. Singlet Oxygen Generation

All the production of singlet oxygen (^1^O_2_) was assessed by the indirect method based on the absorption decay of a solution of 9,10-DMA irradiated in the presence of each extract (*Pm*MW, *Sm*ST, and *Sp*MW), and compared with the decay in the presence of a reference (Zn(II)chorin-*e*_6_ in DMF). In fact, due to its electronic configuration, ^1^O_2_ is an electrophilic agent, reacting preferentially with electron-rich organic molecules and its generation can be monitor easily by the ability of ^1^O_2_ to convert 9,10-DMA in its corresponding endoperoxyde. No photooxidation of 9,10-DMA was observed in the absence of the chlorophyll-rich extracts. However, all the chlorophyll-rich extracts and Zn(II)chorin-*e*_6_ photosensitized 9,10-DMA upon irradiation (Figure S7). According to the results obtained and showed in Figure S7, *Sm*ST produced the highest amount of ^1^O_2_, followed by *Sp*MW and *Pm*MW. In comparison with the reference Znchlorin-*e*_6_, a well-known ^1^O_2_ generator, all extracts produced a lower amount of singlet oxygen under similar conditions. Although the amount of ^1^O_2_ is an important factor, when considering the photosensitizer potential other factors, such as subcellular localization, amphiphilic behavior are parameters that affect the overall efficiency. Once more, the presence of ROS scavenger compounds (xanthophylls and carotenes) seems to interfere in singlet oxygen production. The ^1^O_2_ production ability of the extracts is inversely proportional to the content of these scavengers.

### 2.6. Antibacterial and Antifungal Activity Assays

To evaluate the antimicrobial activity of the ethanol extracts, a dilution agar assay was used. This method is considered one of the standard procedures for the screening of antimicrobial activity of plant extracts [128,129].

Although the effects were always lower than the positive controls, a reduction in colony counts was observed in *L. innocua* and *C. albicans* cultures (Table 3). The extract of *S. patens* was the most active against *L. innocua* (46.9% of photoinactivation), followed by the extracts of *P. maritima* (31.1%) and *S. maritima* (30.1%). Tukey’s test revealed differences in the percent inhibition between the extracts of *S. patens* and either of *P. maritima* or *S. maritima*. No significant differences were found between extracts of *P. maritima* and *S. maritima* (Table 3). Antimicrobial activity is commonly associated with caffeic acids and derivatives [130]. Although *S. patens* is not the species with the larger diversity of hydroxycinnamic acids and derivatives, it is the one with the highest concentrations, (see Table 1), which could explain the results. *P. maritima* extracts were the most active against *C. albicans*. Again, anticandidal activity is also associated with hydroxycinnamic acid derivatives; but, in addition to the concentration of compounds, antimicrobial activity yeasts have been reported to be related to synergic interactions between compounds [131]. The larger diversity of hydroxycinnamic acids was observed in extracts of *P. maritima* and the inhibitory effect against *C. albicans* may be related with these synergic effects. Nevertheless, compared with the positive control (ampicillin and cycloheximide), none of the extracts revealed interesting antimicrobial activity against the tested organisms.

## 3. Materials and Methods

### 3.1. Standards and Reagents

Phosphate buffer saline (PBS) reagents (sodium salt, sodium chloride, potassium chloride, disodium hydrogen phosphate, and potassium dihydrogen phosphate), 2,2-diphenyl-1-picrylhydrazyl (DPPH), 6-hydroxy-2,5,7,8-tetramethylchroman-2-carboxylic acid (trolox), 2,2′-azino-bis(3-ethylbenzothiazoline-6-sulfonic acid (ABTS), trichloroacetic acid (TCA), acetylcholinesterase (AChE) from Electrophorus electricus, 5,5-dithiobis-(2-nitrobenzoic acid), acetylthiocholine iodine, donepezil, and 9,10-dimethylanthracene (DMA) were purchased from Sigma-Aldrich, St. Louis, USA. Potassium persulfate was purchased from J. T. Baker, Pennsylvania, USA; potassium ferricyanide 1% (w/v) was purchased from José M. Vaz Pereira SA, Lisboa, Portugal; and ferric chloride (FeCl_3_) 0.1% (m/v) from HiMedia, Einhausen, Germany. Ethanol, methanol and dimethylformamide (DMF) pro-analysis were used for the extraction and/or dilution of the extracts and acetonitrile, formic acid, and methanol of chromosolv purity were employed in the UHPLC analysis.

Several pure compounds were used as standards to elucidate the identification of the polyphenolic extract’s constituents and also to elaborate the calibration curves, following the external standard method: benzoic acid was purchased from Riedel-de-Haën (Seelze, Germany), whereas ursolic acid, rutin, and quercetin were purchased from Sigma Aldrich (St Louis, USA). Coumaric acid and caffeic acid were supplied by Acros Organics (Geel, Belgium). Kaempferol, gallic and ellagic acids were purchased from TCI (Tokyo, Japan). Oleuropein, (+)-catechin, luteolin, apigenin, quercitrin, isorhamnetin, apigenin-7-*O*-glucoside, luteolin-7-*O*-glucoside, chlorogenic and rosmarinic acids were purchased from EXTRASYNTHESE (Genay Cedex, France).

### 3.2. Plant Collection

To conduct a representative analysis of the species, several specimens of the aerial parts of *S. maritima*, *S. patens*, and *P. maritima* were collected during September 2017 in the saltmarsh areas of Ria de Aveiro (Portugal), and identified by the botanist Helena Silva. *S. maritima* was collected in Gafanha da Encarnação, Ílhavo, 40°37’32.2″ N 8°44’09.4″ W, *S. patens* in Glória, Aveiro, 40°38’01.8″ N 8°39’42.2″ W and *P. maritima* in Aradas, Aveiro, 40°37’18.3″ N 8°39’44.6″ W. A voucher specimen of each *taxon* was deposited in the Herbarium of the Department of Biology, University of Aveiro, Portugal (AVE), under the reference 5427 AVE, 5520 AVE and 5472 AVE, respectively.

### 3.3. Extracts Preparation

To obtain the polyphenolic rich extracts, the aerial parts of *S. maritima*, *S. patens*, and *P. maritima* were washed in running water, pulverized with ethanol 70% (*v*/*v*), and dried in an oven at 65 °C for three days. The resulting material was ground with a grinder and subjected to extraction. Approximately 288 g of *S. maritima*, 233 g of *S. patens*, and 230 g of *P. maritima* dried material was extracted with ethanol pro analysis (3 L, 2 days) using soxhlet, for each independent analysis. The mixture was filtered, and the solvent evaporated using a rotatory vacuum evaporator at 40 °C.

For the chlorophyll-rich extracts, fresh aerial parts of plants of the three taxa were washed in running water and cut into small pieces, being the resulting material subjected to extraction. Approximately, 20 g (for each independent analysis) of plant material was extracted with ethanol p.a. (100 mL) using microwave and ultrasound radiation as well as stirring, at room temperature. The microwave assisted extraction was performed using a MicroSYNTH—Microwave Labstation for synthesis (Milestone, Sorisole, Italy). Microwave radiation was applied for 10 min at 100 °C. For the ultrasound assisted extraction, a Sonorex Digitec (Bandelin, Berlin, Germany) was used. The plant material was treated with ultrasound for 10 min at 30 °C. The room temperature stirring extraction was performed at room temperature under magnetic stirring applied for 48 h. The mixture was filtered, and the solvent evaporated using a rotatory vacuum evaporator.

### 3.4. UHPLC-DAD-ESI/MS^n^ Analysis

For the ultrahigh performance liquid chromatography–mass spectrometry (UHPLC-DAD-ESI/MS^n^) analysis, 100 mg of each polyphenolic extract were dissolved in 5 mL of ethanol (final concentration, 20 mg/mL), and the resulting solutions were filtered through a 0.2 µm Nylon membrane (Whatman). The analysis was carried out using a Thermo Scientific Ultimate 3000RSLC (Dionex) equipped with a Dionex UltiMate 3000 RS diode array detector and coupled to a mass spectrometer. The column used was a thermo scientific hypersil gold column (100 mm × 2.1 mm) with a part size of 1.9 µm and its temperature was maintained at 30 °C. The mobile phase was composed of (A) acetonitrile and (B) 0.1% formic acid (*v*/*v*), both degassed and filtered before use. The flow rate was 0.2 mL/min. The solvent gradient started with 5% of solvent B over 14 min followed by 40% of solvent B for 2 min, 100% over 7 min and finally 5% over 10 min. The injection volume was 2 µL. UV–Vis spectral data were gathered in a range of 250 to 500 nm and the chromatographic profiles were documented at 280 nm. The mass spectrometer used was an LTQ XL linear ion trap 2D equipped with an orthogonal electrospray ion source (ESI). The equipment was operated in negative-ion mode with electrospray ionization source of 5.00 kV and ESI capillarity temperature of 275 °C. The full scan covered a mass range of 50 to 2000 *m*/*z*. Collision-induced dissociation MS/MS and MS^n^ experiments were simultaneously acquired for precursor ions. Three independent analyses were carried out for reproducibility.

For the chlorophyll profile, 20 mg of each chlorophyll-rich extract were dissolved in 2 mL of ethanol (final concentration, 10 mg/mL), and the resulting solutions were filtered through a 0.2 µm Nylon membrane (Whatman). For this analysis a slightly different program was used: The mobile phase was composed of (A) 0.1% formic acid/*v*/*v*) and (B) acetonitrile: methanol (7:30), which were both degassed and filtered before use. The solvent gradient started with 85% of solvent B over 6 min followed by 100% of solvent B for 25 min and finally 85% over 7 min. UV–Vis spectral data were gathered in a range of 430 to 655 nm and the chromatographic profiles were documented at 450 nm. The equipment was operated in positive-ion mode with electrospray ionization source of 4.80 kV.

### 3.5. Identification and Quantification of the Secondary Metabolites

The identification of the individual compounds by UHPLC-MS was achieved by comparing their retention times, UV–Vis spectra, and MS^n^ spectra with data available on the literature. And also with the data of the closest available reference standards, injected under the same UHPL-MS conditions.

The quantification of the individual phenolic compounds in the plant’s polyphenolic extracts was performed by peak integration at 280 nm through the external standard method, using the closest compounds available. A calibration curve was obtained by injection of known concentrations of different standard compounds in order to ensure the quantification of each compound in the samples (Table S4). The results were expressed in mg of compound/100 mg of extract, as mean ± standard deviation of three independent analyses. The analysis of the chlorophyll-rich extracts was solely qualitative.

### 3.6. Evaluation of Antioxidant Activity of the Polyphenolic Rich Extracts

#### 3.6.1. DPPH Radical Scavenging Assay

Research The DPPH radical scavenging assay was carried out following a previously reported procedure [132]. Briefly, 100 µL of ten different concentrations of each plant polyphenolic extract diluted in methanol (500, 250, 125, 62.5, 31.25, 15.63, 7.81, 3.91, 1.95, and 0.98 µg/mL) were prepared and added, in a microplate, to 100 µL of 2,2-diphenyl-1-picrylhydrazyl (DPPH) methanolic solution (0.08 mg/mL). 6-Hydroxy-2,5,7,8-tetramethylchroman-2-carboxylic acid (trolox) was used as standard compound and the same procedure was applied using methanolic solutions of trolox at concentrations of 20.75, 10.38, 5.19, 2.59, 1.30, 0.65, 0.32, 0.16, 0.08, and 0.04 µg/mL. As negative control, a mixture of methanol (100 µL) and DPPH solution (100 µL) was used. Each reaction mixture was added to the 96-well microplate in triplicate. The microplates were placed in the dark at room temperature for 30 min and the absorbance measured at 515 nm on a microplate reader (Synegy HTX multimode microplate reader, BioTek, Vermont, USA) against a blank (absence of DPPH).

The radical-scavenging activity was calculated as a percentage of DPPH discoloration using the following equation; DPPH scavenging effect (%) = [(A_control_ − A_sample_)/A_control_] × 100, where A_control_ = Absorbance of the negative control and A_sample_ = absorbance of the test extract. Based on graphic values of inhibition percentage of DPPH vs. extract concentration, the IC_50_ (concentration of the extract able to inhibit 50% of the DPPH) of each extract was estimated. Three independent assays in triplicate were carried out for reproducibility.

#### 3.6.2. ABTS radical Scavenging Assay

The ABTS^•+^ discoloration assay was performed according to the procedure reported by Catarino et al. [133], with slight modifications. A stock solution of 2,2′-azino-bis(3-ethylbenzothiazoline-6-sulfonic acid (ABTS^•+^) was prepared by reaction of an aqueous solution of ABTS-NH_4_ (7 mM) with 2.45 mM potassium persulfate and stored in the dark at room temperature overnight to allow completed radical generation. The stock solution concentration was adjusted (diluted with methanol) so that its absorbance was ~0.7 at 734 nm. In order to determine the scavenging activity, 100 µL of plant polyphenolic extracts solutions at different concentrations (500, 250, 125, 62.5, 31.25, 15.63, 7.81, 3.91, 1.95, and 0.98 µg/mL, final concentration) and 100 µL of diluted ABTS^•+^ solution were added to a 96-well microplate. Absorbance was measured at 734 nm in a microplate reader (Synegy HTX multimode microplate reader, BioTek, Vermont, USA) against methanol (used as blank) after 20 min of incubation in the dark at room temperature. The same procedure was performed using different concentrations of trolox (20.75, 10.38, 5.19, 2.59, 1.30, 0.65, 0.32, 0.16, 0.08, and 0.04 µg/mL) instead of the extracts. The negative control included the ABTS^•+^ and methanol.

The percentage of inhibition of ABTS^•+^ by the extract was calculated as percentage of ABTS^•+^ discoloration using the following equation: ABTS^•+^ scavenging (%) = [(A_control_ − A_sample_)/A_control_] × 100, where A_control_ = Absorbance of the negative control and A_sample_ = absorbance of the test extract. By plotting the inhibition percentage of ABTS^•+^ against extract concentration, the IC_50_ (concentration of the extract able to inhibit 50% of the ABTS^•+^) of each extract was estimated. Three independent assays in triplicate were carried out for reproducibility.

#### 3.6.3. Ferric Reducing Antioxidant Power (FRAP) Assay

The ability of *S. maritima*, *S. patens*, and *P. maritima*’s polyphenolic extracts to reduce Fe(III) was assessed by the method described by Barreto et al. [132], with slight modifications and performed in a 96-well microplate. For this, 400 µL of extract solutions at concentrations between 500 and 0.98 µg/mL (final concentration) or trolox (standard compound at 0.04–20.75 µg/mL), 400 µL of phosphate buffer (PBS, 0.3 M, pH 6.6) and 400 µL of potassium ferricyanide 1% (w/v) were added to a microtube. The solution was incubated at 50 °C for 20 min. Thereafter, 300 µL of trichloroacetic acid (TCA) 10% (m/v) was added to the previous mixture and centrifuged at 11,000× *g* for 5 min. To each well, 75 µL of the upper layer in each microtube, 75 µL of distillate water and 15 µL of ferric chloride (FeCl_3_) 0.1% (m/v) were added, followed by a vigorous stirring. The absorbance was measured at 700 nm. The mean absorbance values were plotted against concentration, a linear regression analysis was carried out and the EC50 value, corresponding to the extract concentration providing 0.5 of absorbance, was determined. All the assays were performed in triplicate.

#### 3.6.4. Inhibition of Acetylcholinesterase by the Polyphenolic Extracts

Research on the inhibition of acetylcholinesterase by the three extracts was evaluated following a previously reported method [132], with slight modifications. A solution of acetylcholinesterase (AChE) from Electrophorus electricus 0.025 U/mL was prepared in 100 mM PBS solution (pH 7.0), as well as the subtract mixture composed by equal parts of 0.5 mM 5,5-dithiobis-(2-nitrobenzoic acid) and 2.5 mM of acetylthiocholine iodine. The extracts solutions were prepared in sodium phosphate buffer solution (PBS, pH 8) and methanol (1:1) due to solubility issues. Serial doubling dilutions of the extracts were performed in PBS (pH 8) with concentrations ranging from 1 to 0.008 mg/mL. To each of the 96-well microplate, 50 µL of the screened extract and 100 µL of AChE solution (0.025 U/mL) were added. The mixture was incubated for 5 min at 37 °C. Thereafter, 100 µL of the substrate mixture was added. The microplate was placed in the microplate reader programmed to shake for 5 s before each reading and absorbance measured at 415 nm at 0, 150, 300, and 450 s to monitor the reaction rate. Donepezil was used as standard with concentrations ranging from 0.0002 to 0.0261 µg/mL. Reaction rates (v) were calculated using the variation of absorbance from each well with time (v = Abs/∆t). The percentage of inhibition was calculated with the following equation: % of inhibition = 100 − (v_sample_/v_control_) × 100, in which v_sample_ is the reaction rate of the sample and v_control_ the reaction rate of the negative control (absence of inhibitor). The IC_50_ (concentration which causes 50% of inhibition of AChE activity) was calculated by plotting inhibition rates against extract concentration. All the assays were performed in triplicate.

### 3.7. Photophysical Properties of the Chlorophyll-Rich Extracts

#### 3.7.1. Photostability

An aliquot of each ethanolic extract was diluted in 2.5 mL of DMF (absorption of ~1.0 at 411 nm), and then transferred to a quartz cuvette at room temperature under gentle magnetic stirring and irradiated for 5, 15, 35, and 75 min with red light (λ = 630 ± 20 nm) or white light (400–800 nm) for 15 and 30 min. The absorbance spectra were recorded between 350 and 800 nm in the Shimadzu UV-2501 PC spectrometer. UV–Vis spectroscopy assessed the intensity of the Soret band at the different intervals of time and the photostability was expressed as *I_t_*/*I*_0_ (%) (*I_t_* = intensity of the band at given time of irradiation, *I*_0_ = intensity of the band just before the irradiation). The red light system was a home-made LED array composed by a matrix of 5 × 5 LED that takes a total of 25 light sources with an emission peak at 630 nm and a bandwidth at half maximum of 20 nm (irradiance of 10 mW cm^−2^). The white light (400–800 nm) was delivered from a compatible fiber optic probe attached to a 250 W quartz/halogen lamp (LumaCare^®^ model LC122, USA) at an irradiance of 50 mW cm^−2^. Both irradiances were measured with an energy meter Coherent FieldMaxII-Top combined with a Coherent PowerSensPS19Q energy sensor.

#### 3.7.2. Singlet Oxygen Generation

The ability of the extracts to generate singlet oxygen (^1^O_2_) was estimated using 9,10-dimethylanthracene (DMA) as a ^1^O_2_ indicator. For this propose a stock solution of DMA (30 mM), as well as a solution of each extract and of Znchlorin-*e*_6_ in DMF, were prepared. For the ^1^O_2_ generation assay, 2.5 mL of an extract solution in DMF with an absorbance of 0.2 at 410 nm was prepared in a quartz cuvette, and then we added a volume of DMA to reach a final concentration of 10 mM. Then the cuvette was irradiated at 410 nm. Absorption decay of 9,10-DMA at 410 nm was measured at intervals of 2 min up to 10 min. The evaluation of 1O2 was possible since DMA decays in a first order manner during continuous irradiation. Zn(II)chlorin-*e*_6_ was used as reference since it is a known producer of ^1^O_2_. In addition, an 9,10-dimethylanthracene solution was irradiated without extract addition in order to assure that no DMA photodegradation under the experimental conditions occurs. All the assays were performed in triplicate.

### 3.8. Antibacterial and Antifungal Activity Assays

#### 3.8.1. Microbial Strains and Culture Conditions

The antibacterial activity of the extracts from *S. maritima*, *S. patens*, and *P. maritima* were evaluated against Gram-positive (*Listeria innocua* NCTC™ 11288 and *Staphylococcus aureus* ATCC™ 6538) and Gram-negative (*Escherichia coli* ATCC™ 25922) bacteria. The antifungal activity was tested against the yeast *Candida albicans* NCPF 3255 and strains of the molds *Aspergillus niger* and *Lasiodiplodia theobromae* provided by Prof. Cristina Esteves. The bacterial cultures were maintained in Tryptic Soy Agar (TSA, Liofilchem, Roseto degli Abruzzi, Italy). *C. albicans* and *A. niger* were maintained in Potato Dextrose Agar (PDA, Liofilchem), whereas *L. theobromae* was cultivated in Oat Meal Medium containing a filtrate of oat meal broth (15 g/L) and agar (15 g/L). Before each antimicrobial susceptibility assay, fresh cultures were prepared from isolated colonies. For that, bacteria were inoculated in Tryptic Soy Broth (TSB, Liofilchem), and *C. albicans* was inoculated in Potato Dextrose Broth (PDB, Liofilchem). Cultures were incubated overnight, at 37 °C, with agitation (180 rpm). Molds were inoculated in fresh solid media and incubated at 25 °C for 5 days.

#### 3.8.2. Dilution Agar Assay

The antibacterial and antifungal activity of the extracts was determined by the modified agar dilution method. Petri plates were filled with a basal layer (2 mm width) of the appropriate solid medium for each test organism (TSA for bacteria, PDA for *C. albicans*, and *A. niger* and oat meal agar for *L. theobromae*). An overlay prepared by incorporating 500 µL of the plant extract (DMSO solution 0.8 mg/mL) in 4.5 mL of soft medium (0.5% agar) was poured over the basal layer in each plate. As positive controls, the reference antibiotic (ampicillin, Sigma-Aldrich, St. Louis, USA) and antifungal (cycloheximide, Sigma-Aldrich, St. Louis, USA) compounds were used instead of extracts in the same concentrations. An overlay of medium and DMSO without any addition was used as negative control. Three replicates were included in each assay. The fresh cultures of bacteria and *C. albicans* were serially diluted in Ringer solution, and 100 µL aliquots were spread plated on the overlay. Molds were inoculated on the overlay by transferring a mycelium plug (6 mm diameter) to the center of the test plate. The test plates were incubated aerobically at 37 °C for 48 h (*C. albicans*), 37 °C for 24 h (bacteria), 25 °C for 48 h (*Aspergillus*) and 25 °C for 7 days (*L. theobromae*). In the case of bacteria and the yeast, growth was determined by counting the colonies in the replicates of the most suitable dilution, correcting for the dilution factor and aliquot size, averaging the result, and expressing it as CFU/mL. Inhibition was calculated as

% = [(CFU_neg_control_ − CFU_sample_)/CFU_neg_control_)*100] (1)

The growth of molds was determined as the diameter of the mycelium at the end of the incubation. Inhibition was calculated as

% = [(diameter_neg_control_ − diameter_sample_)/diameter_neg_ontrol_)*100] (2)

Three independent assays were performed for each extract.

### 3.9. Statistics

For each polyphenolic extract, three independent analyses were achieved by UHPLC-MS. Kruskal–Wallis one-way analysis of variance on ranks was performed to compare the results obtained in each independent replicate and among the phenolic profile of the species. A Tukey’s test was employed to compare the groups in cases of statistic differences. A *p*-value < 0.05 was considered statistically significant (SigmaPlot 14.0). The quantitative data are expressed as the mean ± standard deviation. A principal component analysis (PCA) was employed in order to detect intergenus and interspecies relationships (Supplementary material). PCA was performed with SatistiXL (StatistiXL, Broadway-Nedlands, AUS) using the polyphenolic profile and the quantitative data as variables.

The results of the biological activities of the polyphenolic extracts were expressed as the mean ± standard deviation and analyzed by ANOVA combined with Tukey’s post hoc test (SigamPlot, 14.0). *p*-values < 5% (*p* < 0.05) were considered to be significant.

The results on photostability and singlet oxygen generation of the chlorophyll-rich extracts are expressed as the mean ± standard deviation of the three independent assays and analyzed by one-way ANOVA or Kruskal–Wallis one-way analysis of variance on ranks combined with Tukey’s post hoc test and Dunn’s test (SigamPlot, 14.0); *p*-value < 5% (*p* < 0.05) was considered significant.

## 4. Conclusions

The interest in halophytes is growing, not only due to their nutritional and/or medicinal value [8], but in an effort to understand their salt tolerance mechanism [134,135,136,137]. It is our hope that more studies regarding saline agriculture, that is, halophyte crop production, are being carried out [134,138]. Nevertheless, considerable researches, to fully understand the matrix effects on halophytes growth, are required. To the best of our knowledge, this study represents the first extensive characterization of polyphenolic richness of the halophytes *S. maritima*, *S. patens*, and *P. maritima* and hopefully will contribute to enlarge the data about halophytes metabolomics. The extracts were particularly rich in caffeic and ferulic acids as well as in trihydroxymethoxyflavone, apigenin and tricin derivatives. There are evidences that salt stress increased the accumulation of flavonoids [139], data that are consistent with our findings. Furthermore, the results highlight the potential of these plants as dietary sources of several compounds with nutraceutical value. Additionally, acetylcholinesterase inhibition and antioxidant activities were also reported for the first time. *S. patens* showed the lowest IC_50_ value for antioxidant activity, which might be related to the exclusive production of some hydroxycinnamic acids and flavones known for their antioxidant properties.

Although, the extracts antimicrobial activity was not interesting, can also be related to their richness in hydroxycinnamic acids and flavones, also known to present antimicrobial activity.

The UHPLC-MS^n^ analysis of the chlorophyll-rich extracts revealed the presence of chlorophyll b; pheophytin a; as well as carotene, lutein, and zeaxanthin. The photostability and the ability to generate singlet oxygen of the chlorophyll-rich extracts were also assessed and the results demonstrate the potential of some of these extracts to be used as photosensitizer agents.

## Figures and Tables

**Table 1:** Identification and quantification of the phenolic compounds in the extracts of *P. maritima* (E*Pm*), *S. maritima* (E*Sm*), and *S. patens* (E*Sp*).

No.	R_t_	[M-H]^−^	MS^2^ (*m*/*z*)	Quantification (mg/100 mg of Extract)			Assigned Identification ^§^
				E*Pm*	E*Sm*	E*Sp*	
***Hydroxycinnamic Acid Derivatives***							
**1**	5.2	353	191 (100), 179 (48), 135 (10)	0.19 ± 0.01	-	-	3-*O*-Caffeoylquinic acid
**2**	7.4	247	179 (100), 161 (40), 135 (15)	-	0.22 ± 0.01	-	Caffeic acid isoprenyl ester
**3**	7.6	341	179 (100), 161(60), 135(51)	-	-	0.20 ± 0.01	Caffeic acid hexoside
**4**	7.9	353	191 (100), 179 (8), 161 (2)	0.20 ± 0.01	-	-	5-*O*-Caffeoylquinic acid
**5**	8.4	367	193 (100), 191 (2), 134 (4)	0.20 ± 0.01	0.22 ± 0.01	0.23 ± 0.01	3-*O*-Feruloylquinic acid
**6**	8.5	429	429 (100), 235 (20), 193 (4), 161 (10)	0.20 ± 0.01	0.22 ± 0.01	0.20 ± 0.01	Isomeric form of feruloyl-caffeoylglycerol
**7**	8.7	429	429 (100), 235 (20), 193 (4), 161 (10)	0.20 ± 0.01	-	-	Isomeric form of feruloyl-caffeoylglycerol
**8**	9.4	655	493 (100), 359 (32), 179 (41)	0.20 ± 0.01	-	-	Salvianolic acid A hexoside
**9**	9.8	431	385 (100), 223 (10), 205 (23)	0.20 ± 0.01	-	-	Sinapic acid hexoside derivative
**10**	10.4	367	193 (80), 173 (100)	0.41 ± 0.14	0.22 ± 0.01	0.24 ± 0.01	4-*O*-Feroluoylquinic acid
**11**	11.9	319	163 (100), 145 (60), 119 (20)	-	-	0.68 ± 0.01	*p*-Coumaroylshikimic acid
**12**	13.0	565	519 (100)	-	0.69 ± 0.01	-	Coumaroylferulic acid hexoside derivative
**13**	13.2	565	519 (100)	0.62 ± 0.01	-	0.64 ± 0.01	Coumaroylferulic acid hexoside derivative
**14**	18.7	443	235 (100), 207 (60), 193 (66), 161 (9), 135 (4)	-	-	0.20 ± 0.01	Diferuloylglycerol
**15**	20.0	613	569 (100), 417 (34), 193 (59)	-	-	0.21 ± 0.01	Ferulic acid guaiacylglyceryl derivative
***Flavonoids***							
**16**	10.2	653	635 (65), 445 (100), 313 (8)	0.46 ± 0.01	0.50 ± 0.01	-	Trihydroxymethylenedioxyflavone-*O*-pentosyl-*O*-hydroxyferuloyl
**17**	10.5	447	357 (64), 327 (100)	0.60 ± 0.02	0.65 ± 0.02	0.74 ± 0.04	Luteolin-8-*C*-hexoside
**18**	10.8	563	545 (2), 473 (100), 443 (69), 383 (22), 353 (28)	0.50 ± 0.01	0.55 ± 0.01	-	Apigenin-6(8)-*C*-hexoside-8(6)-*C*-pentoside
**19**	10.9	461	371 (42), 341 (100), 313 (32), 299(4)	-	-	0.80 ± 0.05	Trihydroxymethoxy flavone *C*-hexoside (isomer I)
**20**	11.1	593	473 (44), 383 (100), 353 (70)	-	1.09 ± 0.13	0.48 ± 0.01	Apigenin di-*C*-hexoside
**21**	11.4	623	503 (10), 443 (100), 353 (4), 323 (26)	-	0.79 ± 0.08	-	Dihydroxymethoxy flavone caffeoyl *C*-hexoside
**22**	11.5	533	515 (24), 473 (63), 443 (100), 383 (14), 353 (14)	0.46 ± 0.01	-	-	Apigenin-di-*C*-pentoside
**23**	11.6	431	269 (100)	0.50 ± 0.01	3.14 ± 0.24	0.56 ± 0.02	Apigenin-*O*-hexoside
**24**	11.8	607	487 (41), 443 (100), 353 (40), 323 (26)	-	-	0.50 ± 0.01	Dihydroxymethoxyflavone coumaroyl-*C*-hexoside
**25**	12.0	461	443 (6), 371 (21), 341 (100),	0.47 ± 0.01	1.54 ± 0.14	0.51 ± 0.01	Trihydroxymethoxy flavone *C*-hexoside (isomer II)
**26**	12.2	447	429 (24), 357 (80), 327 (100), 285 (10)	0.48 ± 0.01	-	-	Luteolin *C*-hexoside
**27**	12.3	461	371 (8), 341 (100)	-	0.53 ± 0.01	-	Trihydroxymethoxy flavone *C*-hexoside (isomer III)
**28**	12.5	815	507 (100)	-	-	0.47 ± 0.01	Hydroxydimethoxydimethyl homoisoflavone I ^♣^
**29**	12.6	769	593 (70), 413 (100), 323 (6), 293 (52)	-	0.51 ± 0.01	-	Apigenin *C*-hexoside-*O*-caffeoylglucoronide
**30**	12.8	537	493 (10), 375 (100), 331 (6)	0.49 ± 0.01	-	0.48 ± 0.01	Biapigenin (Amentoflavone)
**31**	13.4	491	476 (9), 329 (100)	0.47 ± 0.01	-	-	Tricin-7-*O*-hexoside
**32**	13.5	431	341 (28), 311 (100)	-	0.55 ± 0.01	-	Apigenin-6-*C*-hexoside
**33**	13.9	461	371 (8), 341 (100)	-	0.52 ± 0.01	-	Trihydroxymethoxy flavone *C*-hexoside (isomer IV)
**34**	14.1	687	525 (100), 329 (8)	0.46 ± 0.01	-	-	Tricin guaiacylglyceryl hexoside (isomer I)
**35**	14.3	431	341 (10), 311 (100)	-	0.55 ± 0.01	-	Apigenin-8-*C*-hexoside
**36**	14.6	687	525 (100), 329 (8)	0.46 ± 0.01	-	-	Tricin guaiacylglyceryl hexoside (isomer II)
**37**	14.7	461	371 (8), 341 (100)	-	0.51 ± 0.01	-	Trihydroxymethoxy flavone *C*-hexoside (isomer V)
**38**	15.0	373	329 (100) [tricin-H]^−^	-	0.51 ± 0.01	-	3-*O*-Acetyl tricin
**39**	15.1	611	593 (18), 449 (100)	-	-	0.47 ± 0.01	Erioctioyl di-*O*-hexoside
**40**	15.2	409	329 (100)	-	0.51 ± 0.01	-	Tricin sulfate
**41**	15.6	629	611 (6), 467 (100)	-	-	0.47 ± 0.01	Trihydroxymethylenedioxyflavone derivative ^♥^
**42**	16.0	605	329 (100), 314 (10)	-	0.51 ± 0.01	0.47 ± 0.01	Tricin guaiacylglycerylsulfate
**43**	16.4	403	388 (9), 329 (100)	-	0.54 ± 0.01	0.49 ± 0.01	Tricin glyceryl
**44**	16.5	507	492 (100), 339 (12), 311 (8)	-	0.50 ± 0.01	0.48 ± 0.01	Hydroxydimethoxydimethyl homoisoflavone II ^♦^
**45**	16.7	691	497 (100), 329 (60), 314 (10)	0.58 ± 0.01	0.58 ± 0.01	-	Tricin *C*-methylgalloyl glucuronide
**46**	16.8	417	373 (10), 354 (21), 329 (100)	Tr	-	-	Tricin derivative
**47**	16.9	643	481 (100), 329 (20), 314 (12), 299 (10)	-	-	0.48 ± 0.01	Tricin-*C*-galloyl-*O*-hexoside
**48**	17.6	329	314 (100), 299 (7)	0.48 ± 0.01	0.79 ± 0.02	0.62 ± 0.02	Tricin
**49**	17.9	587	571 (87), 439 (100), 359 (16)	0.47 ± 0.01	0.77 ± 0.02	-	Quercetagetin trimethyl *O*-sulfate pentoside
**50**	18.0	525	329 (100)	-	-	0.58 ± 0.02	Tricin-4-*O*-guaiacylglyceryl
**51**	18.2	541	495 (100), 345 (19)	-	0.55 ± 0.01	0.50 ± 0.01	Spinacetin guaiacylglyceryl
**52**	19.1	569	551 (34), 345 (100)	-	0.49 ± 0.01	0.48 ± 0.01	Spinacetin sinapoyl
**53**	19.9	853	805 (12), 493 (100), 329 (54), 314 (13), 299 (12)	-	0.50 ± 0.01	0.48 ± 0.01	Tricin secoisolariciresinol coumaroyl
**54**	20.1	817	577 (60), 559 (88), 537 (42), 451 (46), 407 (100)	-	-	0.52 ± 0.01	Procyanidyn dimer derivative
**55**	20.4	599	447 (100), 285 (47)	-	0.52 ± 0.01	0.51 ± 0.01	Kaempferol galloyl hexoside
**56**	20.5	599	584 (100), 419 (16), 283 (18)	0.46 ± 0.01	0.53 ± 0.01	0.52 ± 0.01	Methylgnistein caffeoyl derivative
**57**	21.0	641	623 (100), 445 (32), 293 (30), 255 (21)	-	-	0.49 ± 0.01	Dihydroxyflavanone Acetyl guaiacylglyceryl galloyl
**58**	21.7	577	415 (22), 299 (100)	0.48 ± 0.01	-	0.48 ± 0.01	Trihydroxymethoxyflavone-*O*-hexose-*O*-malloyl
**59**	22.1	649	603 (10), 487 (51), 413 (82), 299 (100)	0.45 ± 0.01	-	-	Trihydroxymethoxy flavone derivative ^♠^
**60**	22.4	540	480 (100), 304 (9)	-	0.50 ± 0.01	-	*O*-Methylcatechin acetate glucuronide
***Others***							
**61**	2.8	241	197 (100)	2.36 ± 0.35	2.23 ± 0.12	0.53 ± 0.02	Syringic acid derivative
**62**	9.5	535	197 (21), 163 (8), 129 (10), 85 (100)	0.19 ± 0.01	-	-	Coumaroylsyringylglucarate acid
**63**	12.9	683	521 (42), 359(27), 329 (100)	Tr	-	Tr	Lariciresinol dihexoside
**64**	14.8	231	213 (100), 187(10)	-	-	Tr	Costunolide
**65**	18.4	571	525 (100)	Tr	0.09 ± 0.01	0.06 ± 0.01	Dehydrated oleanolic acid pentoside

Tr = traces, Rt = Retention time in min., [M-H]^−^ = pseudomolecular (*m*/*z*) and MS^2^ = fragment ions (relative peak intensities), ^§^ Identification based on spectroscopic data (UV–Vis and MS), fragmentation pattern (Table S3), comparing with pure standards and/or pure similar derivatives, ^♣^ Hydroxydimethoxydimethyl homoisoflavone *O*-coumaroylhexoside-*C*-methylgalloyl, ^♥^ Trihydroxymethylenedioxyflavone dihydrogalloyl hexoside, ^♦^ Hydroxydimethoxydimethyl homoisoflavone-*C*-methylgalloyl, ^♠^ Trihydroxymethoxy flavone *O*-glycosylhexoside-*O*-5-hydroxy-4-mehoxypentanoic acid.

**Table 2:** Retention times, ultraviolet–visible (UV–Vis) absorption maxima, pseudomolecular ion [M+H]^+^, and identification of the major peaks uncovered.

R_t_	[M+H]^+^	UV–Vis	*Pm*MW *	*Pm*US *	*Sm*MW *	*Sm*ST *	*Sm*US *	*Sp*MW *	*Sp*ST *	*Sp*US *	Assigned Identification
8.8	569	431, 443, 473	57.2	14.8	9.3	23.9	9.9	27.4	15.5	1.2	Lutein
17.5	885	420, 435, 460sh, 652	15.8	12.5	12.4	13.1	18.8	6.5	0.6	7.0	Chlorophyll *b* I
18.0	889	435, 651	-	-	3.4	2.5	3.6	-	-	-	Chlorophyll *b* II
18.4	569	438sh, 455, 476	17.4	3.7	-	-	-	-	-	-	Zeaxanthin
18.8	537	273, 453	-	-	18.5	12.6	6.3	13.8	36.8	-	Carotene
19.8	887	407, 655	-	-	-	-	-	15.0	35.4	-	Chlorophyll *b* III
20.5	871	410, 653, 665	7.6	59.8	39.6	40.7	51.6	32.4	10.4	91.8	Pheophytin *a* I
21.2	871	420, 438, 455, 663	1.9	9.6	16.9	7.2	9.7	4.8	1.4	-	Pheophytin *a* II

* The values are expressed as peak area percentage. Rt = Retention time in min.; [M+H]^+^ = pseudomolecular ion in *m*/*z*; UV–Vis data in nm; *Pm*MW = microwave extract of *P. maritima*; *Pm*US = ultrasound extract of *P. maritima*; *Sm*MW = microwave extract of *S. maritima*; *Sm*ST = room temperature extract of *S. maritima*; *Sm*US = ultrasound extract of *S. maritima*; *Sp*MW = microwave extract of *S. patens*; *Sp*ST = room temperature extract of *S. patens*; *Sp*US = ultrasound extract of *S. patens*.).

**Table 3:** Percentage of inhibition of *S. maritima*, *S. patens*, and *P. maritima* ethanol extracts against *C. albicans* and *L. innocua* relatively to the untreated control.

Extract	*P. maritima*	*S. maritima*	*S. patens*
***C. albicans***	31.06 ± 6.56 *	30.54 ± 6.17 *	46.90 ± 8.30 *
***L. innocua***	44.59 ± 0.12 *	41.59 ± 3.76 *	31.89 ± 1.94 *

All extracts showed statistically significant differences relatively to the positive control; * Statistically different relatively to S. patens (Tukey’s test), (*p* < 0.05).

## Supplementary Materials

The following are available online. Figure S1: Score plot of the PC 1 *vs* PC2, Table S1: Explained variance along the three first axes of PCA, Table S2: Loadings between compounds and the principal components axis, Figure S2: UHPLC-MS chromatogram of (A) *P. maritima*, (B) *S. maritime*, and (C) *S. patens*, recorded at 280 nm, Table S3: Additional data of the identified compounds in the studied taxa, Figure S3: Structure of salvianolic acid A (8) and its main fragments, Figure S4: Structure of some flavonoids identified in the polyphenolic extracts of the three studied taxa, Table S4: Linearity (y = mx + b, where y corresponds to the standard peak area and x corresponds to the mass of standard), LOD and LOQ of pure compounds used as reference, Table S5: Chlorophyll a (Cha), b (Chb) and total (Cha+b) content (mg/100 mg of extract) of *P. maritima*’s ethanol extracts from microwave (PMMW), room temperature stirring (PMST) and ultrasound (PMUS) as well as *S. maritima* microwave (SMMW), room temperature stirring (SMST), and ultrasound (SMUS) extracts and *S. patens* microwave (SPMW), room temperature stirring (SPST), and ultrasound (SPUS) extracts, Figure S5: Chlorophyll a (Cha), b (Chb) and total (Cha+b) content (mg/100 mg of extract) of *P. maritima*’s ethanol extracts from microwave (PMMW), room temperature stirring (PMST) and ultrasound (PMUS) as well as *S. maritima* microwave (SMMW), room temperature stirring (SMST) and ultrasound (SMUS) extracts and *S. patens* microwave (SPMW), room temperature stirring (SPST) and ultrasound (SPUS) extracts, Table S6: Extraction yield (%) of *P. maritima*’s ethanol extracts from microwave (PMMW), room temperature stirring (PMST), and ultrasound (PMUS); *S. maritima* microwave (SMMW), room temperature stirring (SMST), and ultrasound (SMUS) extracts; and *S. patens* microwave (SPMW), room temperature stirring (SPST), and ultrasound (SPUS) extracts, Figure S6: Extraction yield (%) of *P. maritima*’s ethanol extracts from microwave (PMMW), room temperature stirring (PMST), and ultrasound (PMUS) as well as *S. maritima* microwave (SMMW), room temperature stirring (SMST), and ultrasound (SMUS) extracts and *S. patens* microwave (SPMW), room temperature stirring (SPST), and ultrasound (SPUS) extracts, Table S7: Antioxidant capacity and acetylcholinesterase inhibitory activity of *P. maritima*, *S. maritima*, and *S. patens* ethanol extracts, Figure S7: Graphical representation of ln (A_0_/A) in function of time(s) of positive control (Znchlorin-*e*_6_, negative control, and of the extracts SMST, SPMW, and PMMW, Table S8: Percentage of photodegradation in PMMW, SMST and SPMW after incidence for 5, 15, 35 and 75 min with red light (630 ± 20 nm, 10 mW cm^−2^) and for 15 and 30 min with white light (400-800 nm, 50 mW cm^−2^).
