# Supplementary material for: Puccinellia maritima, Spartina maritime, and Spartina patens Halophytic Grasses: Characterization of Polyphenolic and Chlorophyll Profiles and Evaluation of Their Biological Activities
Source: Molecules. 2019 Oct 22;24(20):3796. doi: 10.3390/molecules24203796 (PMC6832526; doi:10.3390/molecules24203796)
Supplement: Supplementary file 1 [file molecules-24-03796-s001.pdf]

Supplementary material

Puccinellia maritima, Spartina maritima and *Spartina patens* halophytic grasses: characterization of polyphenolic and chlorophyll profiles and evaluation of their biological activities

Maria V. Faustino,[a] Maria A. F. Faustino,[a] Helena Silva,[b] Ângela Cunha,[b] Artur M. S. Silva [a] and Diana C. G. A. Pinto*[a]

[a]QOPNA & LAQV-REQUIMTE, Department of Chemistry, University of Aveiro, Campus de Santiago, 3810-193 Aveiro, Portugal.
[b]Department of Biology & CESAM, University of Aveiro, Campus de Santiago, 3810-193 Aveiro, Portugal.

*Corresponding author
E-mail address: **diana@ua.pt**

Contents

1. Principal component analysis 3S

Score plot of the PC 1 *vs* PC2 .... 4S

Loadings between compounds and the principal components axis 5S

2. UHPLC-MS chromatograms recorded at 280 nm 7S

3. Total phenolic compounds quantification by Folin-Ciocalteu method 8S

4. Additional data of the identified compounds in the studied *taxa* 9S

5. Salvianolic acid A main fragments 13S

6. Structure of some flavonoids identified in the polyphenolic extracts of the three studied *taxa* 13S

7. Extraction yield of the polyphenolic extracts 13S

8. Total chlorophyll quantification 14S

9. Extraction yield of the chlorophyll rich extracts 16S

10. Biological activity results 18S

11. Photophysical properties of the chlorophyll rich extracts' 19S

References 20S

1. Principal component analysis

Aiming to characterize the different studied *taxa* thought their polyphenolic profile and to recognize the key compounds for their discrimination, a principal component analysis (**PCA**) was employed. This multivariate statistical method has been used as a basis for examining intraspecies variation, since it elicits and displays dissimilarities among variables.[1,2]

The **PCA** was completed with 62 compounds and their respective content (compounds **42**, **62** and **63** were excluded from the analysis due to its extremely low concentration). The first two components account for 98.62% of the total variance since PC1 explains 53.35% of the variance and PC2 44.92%. PC3 only account for 0.8% of the total variance (**Table S1**). Therefore, the analysis of the data was achieved by using the scatterplot of PC1 *vs* PC2 which allowed an efficient separation of the three studied *taxa* explaining the highest amount of variance. The first component showed high positive loading (> 0.9) for 12 compounds (**1**, **4**, **7-9**, **22**, **26**, **31**, **34**, **36**, **59** and **65**) and high negative loadings (<-0.9) also for 12 compounds (**5**, **20**, **42-44**, **48**, **51-53**, **55**, **56** and **62**). PC2 has high positive loadings for 13 compounds (**3**, **11**, **14**, **15**, **19**, **24**, **28**, **39**, **41**, **47**, **50**, **54** and **57**) and high negative loadings for 5 compounds (**16**, **18**, **45**, **49** and **61**) (**Table S2**). On the overall, 64.6% of the identified compounds contribute to distinguish the two species. Furthermore, through comparison of the score plot (**Figure S1**) and **table 1**, it was evident that the compounds whose vectors point towards each species are exclusively produced by that species. This is only an exception for compounds **23**, **25** and **60**, which are produced in higher quantities by *S. maritima* comparing to the other species.

Puccinellia maritima appears isolated in the positive quadrant of PC1 and negative of PC2, reflecting the presence of compounds **1**, **4**, **7-9**, **22**, **26**, **31**, **34**, **36**, **59** which were exclusively produced by this species (**Figure S1**). Compounds **1** to **9** are hydroxycinnamic acid derivatives, which can be relevant since *P. maritima* was the species with higher content of these molecules. Nonetheless, the remaining compounds, especially the tricin derivatives (**31**, **34** and **36**) seem to contribute the most for the separation since are produced in higher quantities.

Spartina maritima is located in the negative quadrant of PC1 and PC2, mainly due to the presence of compounds **27**, **33**, **37** (trihydroxymethoxyflavone derivatives), **29**, **32** (apigenin derivatives), **38** and **40** (tricin derivatives), exclusively produced by this species (**Figure S1**). Additionally, compound **23** also seem to play an important role in the separation of these *taxa* since its production is six times higher compared to the other studied species (**Table 1**).

At last, *S. patens* appear positioned in the negative quadrant of PC1 and positive of PC2, mostly influenced by the compounds **19** and **41** (trihydroxymethoxyflavone derivatives) since they are produced in higher quantities and exclusively by this species. Additionally, *S. patens* was the only, among the three studied *taxa*, to produce a flavanone (compound **39**) which also contributed for the separation on the score plot.

The **PCA** analysis revealed an evident variation of the phenolic composition among the studied species. The qualitative and quantitative variables allowed a clear distinction among the investigated taxa that can be

attributed to the exclusive production of key compounds by each independent species. In the case of *P. maritima*, the abundance and diversity of hydroxycinnamic acids clearly allowed a differentiation between this *taxon* and the ones from *Spartina* genus. The differences among *S. maritima* and *S. patens* rely mainly on the flavone profile which plays the most important role in their distinction. It is interesting that a clear differentiation among the two studied *Spartina* species were achieved, which can indicate flavones as possible chemotaxonomic markers. Nevertheless, we strongly suggest the analysis of a larger number of samples to accurately infer the success of this chemotaxonomic approach.

Table S1. Explained variance along the three first axes of **PCA**.

Axis	Eigenvalue	Percentage of variance explained (%)	Cumulative percentage of variance explained (%)
PC1	33.074	53.345	53.345
PC2	27.848	44.916	98.262
PC3	0.507	0.818	99.079

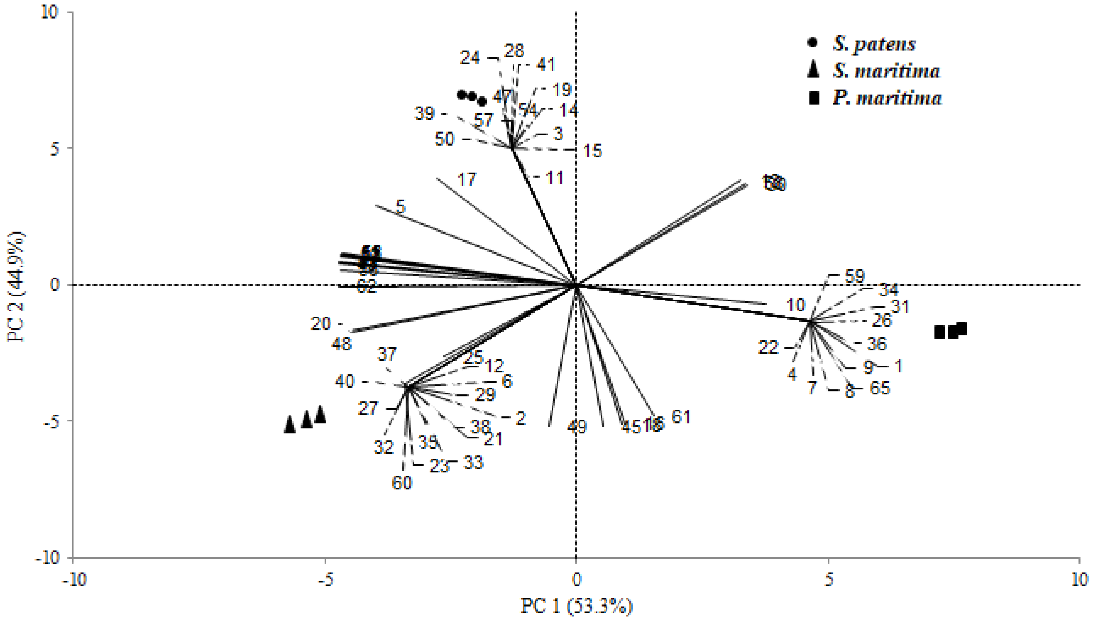

Fig. S1. Score plot of the PC 1 *vs*. PC2 from a **PCA** performed with 62 variables (each number corresponds to an identified compound from **table 1**) of *S. maritima*, *S. patens*, and *P. maritima*.

Table S2. Loadings between compounds and the principal components axis.

Variable	PC 1	PC 2	PC 3
**1**	0.967	-0.250	0.014
**2**	-0.701	-0.713	-0.025
**3**	-0.267	0.963	0.008
**4**	0.968	-0.251	0.017
**5**	-0.832	0.554	-0.010
**6**	-0.701	-0.713	-0.025
**7**	0.968	-0.251	0.017
**8**	0.968	-0.250	0.019
**9**	0.968	-0.250	0.019
**10**	0.786	-0.134	0.087
**11**	-0.267	0.964	0.009
**12**	-0.701	-0.713	-0.008
**13**	0.681	0.732	0.024
**14**	-0.267	0.963	0.008
**15**	-0.267	0.963	0.008
**16**	0.203	-0.979	0.002
**17**	-0.577	0.744	0.190
**18**	0.187	-0.982	-0.002
**19**	-0.267	0.962	0.013
**20**	-0.937	-0.333	0.091
**21**	-0.699	-0.710	0.072
**22**	0.968	-0.250	0.016
**23**	-0.715	-0.696	0.033
**24**	-0.267	0.964	0.009
**25**	-0.548	-0.508	0.658
**26**	0.968	-0.250	0.018
**27**	-0.701	-0.713	-0.017
**28**	-0.267	0.963	0.008
**29**	-0.701	-0.713	-0.017
**30**	0.709	0.705	0.027
**31**	0.968	-0.251	0.017
**32**	-0.700	-0.713	-0.032
**33**	-0.701	-0.713	-0.025
**34**	0.968	-0.250	0.018
**35**	-0.701	-0.713	-0.028
**36**	0.968	-0.251	0.017
**37**	-0.701	-0.713	-0.017
**38**	-0.701	-0.713	-0.017
**39**	-0.267	0.963	0.008
**40**	-0.701	-0.713	-0.017
**41**	-0.267	0.964	0.009

**42**	-0.979	0.205	-0.009
**43**	-0.988	0.152	-0.010
**44**	-0.977	0.210	-0.018
**45**	0.108	-0.994	-0.007
**47**	-0.267	0.964	0.009
**48**	-0.944	-0.323	-0.017
**49**	-0.118	-0.993	-0.016
**50**	-0.267	0.964	0.010
**51**	-0.987	0.159	-0.010
**52**	-0.974	0.227	-0.009
**53**	-0.976	0.216	-0.017
**54**	-0.267	0.964	0.010
**55**	-0.973	0.229	-0.016
**56**	-0.982	0.105	-0.031
**57**	-0.267	0.963	0.008
**58**	0.701	0.713	0.025
**59**	0.968	-0.251	0.017
**60**	-0.701	-0.713	-0.025
**61**	0.319	-0.924	-0.017
**62**	-0.988	-0.008	-0.029
**65**	0.968	-0.251	0.017

2. UHPLC-MS chromatograms recorded at 280 nm

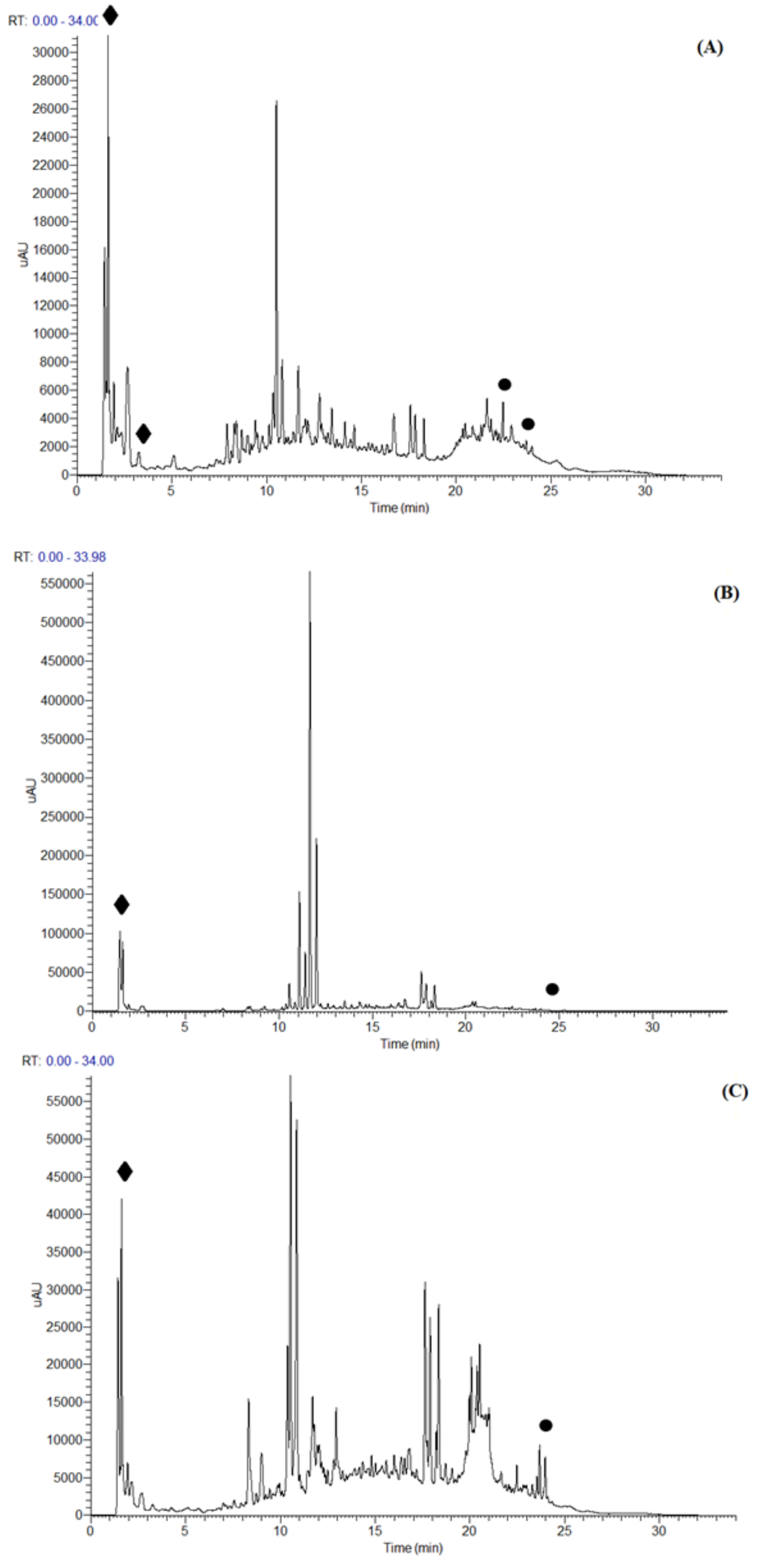

Fig. S2. UHPLC-MS chromatogram of (A) *P. maritima*, (B) *S. maritima* and (C) *S. patens*, recorded at 280 nm (♦ solvent, ● chlorophylls).

3. Total phenolic compounds quantification by Folin-Ciocalteu method

Aiming to determine the total phenolic content in the studied plants' extracts and to compare the values obtained with the ones achieved by UHPLC-MS, the Folin-Ciocalteu method was employed. In a 96 well-microplate, 15 µL of each extract was added to 15 µL of Folin-Ciocalteu reagent and 60 µL of milliQ water. After 5 minutes, 150 µL of the Na_2CO_3 solution was added. The mixture was homogenized and incubated in the dark for 60 minutes at 30 ºC. The absorbance was measured at 700 nm and the amount of total phenolic compounds was expressed as gallic acid equivalent (mg GAE/g of extract). For this, a calibration curve was performed with gallic acid.

This determination allowed to confirm that *S. maritima*'s extract is the one with more content in phenolic compounds (76.55 ± 1.06 mg GAE/g of extract), followed by *S. patens* (71.48 ± 1.36 mg GAE/g) and *P. maritima* (43.78 ± 2.17 mg GAE/g). Nonetheless, the concentrations attained through this colorimetric method were several times lower than the ones achieved by UHPLC-MS. In this case, the milligrams of phenolic compounds per gram of extract were 133, 231 and 157.7 in *P. maritima*, *S. maritima*, and *S. patens*, respectively. It is obvious that the total phenolic content measured by the Folin-Ciocalteu procedure does not give the full picture of the real quantity of the phenolic constituents in the extracts. The quality and diversity of the phenolics present are also not taken into consideration.[3] Therefore, Folin-Ciocalteu method was only useful for an estimation of the phenolic content and cannot be used for a reliable and accurate quantification.

Table S3. Additional data of the identified compounds in the studied *taxa*.
R_t=Retention time in min., λ_{max}=wavelength of maximum absorption in the UV-Vis region, $[M-H]^-$ =pseudomolecular and MS^2=fragment ions (relative peak intensities) [type of fragment and identification] and some MS^3 fragment ions.

Nr.	Rt (min)	λ_{max} (nm)	$[M-H]^-$ (*m/z*)	MS^2 (*m/z*)	Assigned identification
				Hydroxycinnamic acid derivatives	
1	5.2	246; 328	353	191 (100) $[quinic\ acid-H]^-$<br>179 (48) $[caffeic\ acid-H]^-$<br>135 (10) $[caffeic\ acid-H-CO_2]^-$	3-*O*-caffeoylquinic acid
2	7.4	239, 305	247	179 (100) $[caffeic\ acid-H]^-$<br>161 (40) $[caffeic\ acid-H-H_2O]^-$<br>135 (15) $[caffeic\ acid-H-CO_2]^-$	Caffeic acid isoprenyl ester
3	7.6	232; 329	341	179 (100) $[caffeic\ acid-H]^-$<br>161(60) $[caffeic\ acid-H-H_2O]^-$<br>135(51) $[caffeic\ acid-H-CO_2]^-$	Caffeic acid hexoside
4	7.9	238, 323	353	191 (100) $[quinic\ acid-H]^-$<br>179 (8) $[caffeic\ acid-H]^-$<br>161 (2) $[caffeic\ acid-H-H_2O]^-$	5-*O*-Caffeoylquinic acid
5	8.4	240, 323	367	193 (100) $[ferulic\ acid-H]^-$<br>191 (2) $[quinic\ acid-H]^-$<br>134 (4) $[ferulic\ acid-H-CO_2-CH_3]^-$	3-*O*-Feruloylquinic acid
6	8.5	290, 323	429	429 (100) $[M-H]^-$<br>235 (20) $[M-H-ferulic\ acid]^-$<br>193 (4) $[ferulic\ acid-H]^-$<br>161 (10) $[caffeic\ acid-H-H_2O]^-$	Isomeric form of feruloyl-caffeoylglycerol
7	8.7	290, 323	429	429 (100) $[M-H]^-$<br>235 (20) $[M-H-ferulic\ acid]^-$<br>193 (4) $[ferulic\ acid-H]^-$<br>161 (10) $[caffeic\ acid-H-H_2O]^-$	Isomeric form of feruloyl-caffeoylglycerol
8	9.4	276, 338	655	493 (100) $[salvianolic\ acid\ A-H]^-$<br>359 (32) $[C_{18}H_{15}O_8-H]^-$<br>179 (41) $[caffeic\ acid-H]^-$	Salvianolic acid A hexoside
9	9.8	242, 314	431	385 (100) [sinapic acid hexoside-H]-,<br>223 (10) [sinapic acid-H]-<br>205 (23) $[sinapoyl-H]^-$	Sinapic acid hexoside derivative
10	10.4	238, 325	367	193 (80) $[Ferulic\ acid-H]^-$<br>173 (100) $[quinic\ acid-H-H_2O]^-$	4-*O*-Feroluoylquinic acid
11	11.9	239, 325	319	163 (100) $[coumaric\ acid-H]^-$<br>145 (60) $[coumaric\ acid-H-H_2O]^-$<br>119 (20) $[coumaric\ acid-H-CO_2]^-$	*p*-Coumaroylshikimic acid
12	13.0	243, 275	565	519 (100) $[M-H-H_2O-CO]^-$<br>MS^3[519]:<br>357 (100) $[M-H-hexose]^-$<br>193 (30) $[ferulic\ acid-H]^-$	Coumaroylferulic acid hexoside derivative
13	13.2	245, 279	565	519 (100) $[M-H-H_2O-CO]^-$<br>MS^3[519]:<br>357 (100) $[M-H-hexose]^-$<br>193 (30) $[ferulic\ acid-H]^-$	Coumaroylferulic acid hexoside derivative
14	18.7	250, 335	443	235 (100) $[C_{12}H_{13}O_5-H]^-$<br>207 (60) $[C_{11}H_{11}O_4-H]^-$<br>193 (66) $[M-H-ferulic\ acid]^-$<br>161 (9) $[C_{10}H_9O_2-H]^-$<br>135 (4) $[C_8H_8O_2-H]^-$	Diferuloylglycerol
15	20.0	244, 329	613	569 (100) $[M-H-CO_2]^-$<br>417 (34) $[M-H-guaiacylglyceryl]^-$<br>193 (59) $[ferulic\ acid-H]^-$	Ferulic acid guaiacylglyceryl derivative
				Flavonoids	
16	10.2	271, 345	653	635 $[M-H-H_2O]^-$<br>445 (100) $[\ M-H-\ H_2O-hydroxyferuloyl]^-$<br>313 $[trihydroxymethylenedioxyflavone-H]^-$	Trihydroxymethylenedioxyflavone-*O*-pentosyl-*O*-hydroxyferuloyl
17	10.5	269, 345	447	357 (64) $[M-H-90]^-$<br>327 (100) $[M-H-120]^-$	Luteolin-*C*-hexoside
18	10.8	271, 338	563	545 (2) $[M-H-60]^-$<br>473 (100) $[M-H-90]^-$	Apigenin-8-*C*-hexoside-6-*C*-pentoside or Apigenin-6-*C*-hexoside-8-*C*-pentoside

				443 (69) [M-H-120]$^-$<br>383 (22) [M-H-120-60]$^-$<br>353 (28) [M-H-120-90]$^-$	
19	10.9	269, 346	461	371 (42) [M-H-90]$^-$<br>341 (100) [M-H-120]$^-$<br>313 (32) [M-H-CO]$^-$<br>299(4) [trihydroxymethoxy flavone-H]$^-$<br>MS^3[341]:<br>299 (100) [trihydroxymethoxy flavone-H]$^-$	Trihydroxymethoxy flavone *C*-hexoside (isomer I)
20	11.1	270, 337	593	473 (44) [M-H-120]$^-$<br>383 (100) [Ag + 113]$^-$<br>353 (70) [Ag+83]$^-$	Apigenin di-*C*-hexoside
21	11.4	270, 350	623	503 (10) [M-H-120]$^-$<br>443 (100) [M-H-caffeoyl]$^-$<br>353 (4) [Ag + 71]$^-$<br>323 (26) [Ag + 41]$^-$	Dihydroxymethoxy flavone caffeoyl *C*-hexoside
22	11.5	275, 339	533	515 (24) [M-H-H_2O]$^-$<br>473 (63) [M-H-60]$^-$<br>443 (100) [M-H-90]$^-$<br>383 (14) [Ag+113]$^-$<br>353 (14) [Ag+83]$^-$	Apigenin-di-*C*-pentoside
23	11.6	262, 328	431	269 (100) [apigenin-H]$^-$	Apigenin-*O*-hexoside
24	11.8	240, 334	607	487 (41) [M-H-120]$^-$<br>443 (100) [M-H-coumaroyl]$^-$<br>353 (40) (4) [Ag + 71]$^-$<br>323 (26) [Ag + 41]$^-$	Dihydroxymethoxyflavone coumaroyl-*C*-hexoside
25	12.0	270, 350	461	443 (6) [M-H-H_2O]$^-$<br>371 (21) [M-H-90]$^-$<br>341 (100) [M-H-120]$^-$<br>MS^3[341]:<br>313 (100) [M-H-CO]$^-$<br>299 (60) [Trihydroxymethoxy flavone-H]$^-$	Trihydroxymethoxy flavone *C*-hexoside (isomer II)
26	12.2	265, 350	447	429 (24) [M-H-H_2O]$^-$<br>357 (80) [M-H-90]$^-$<br>327 (100) [M-H-120]$^-$<br>285 (10) [luteolin-H]$^-$	Luteolin *C*-hexoside
27	12.3	268, 347	461	371 (8) [M-H-90]$^-$<br>341 (100) [M-H-120]$^-$<br>MS^3[341]:<br>313 (100) [M-H-CO]$^-$<br>299 (54) [Trihydroxymethoxy flavone-H]$^-$	Trihydroxymethoxy flavone *C*-hexoside (isomer III)
28	12.5	275, 336	815	507 (100) [M-H- coumarylhexose]$^-$<br>MS^3[507]:<br>339 (100) [Hydroxydimethoxydimethyl homoisoflavone -H]$^-$<br>311 (2) [Hydroxydimethoxydimethyl homoisoflavone -H-CO]$^-$	Hydroxydimethoxydimethyl homoisoflavone *O*-coumaroylhexoside-*C*-methylgalloyl
29	12.6	274, 331	769	593 (70) [M-H-glucuronide]$^-$<br>413 (100) [M-H- caffeoylglucuronide]$^-$<br>323 (6) [M-H-caffeoylglucuronide-90]$^-$<br>293 (52) [M-H-caffeoylglucuronide-120]$^-$	Apigenin *C*-hexoside-*O*-caffeoylglucoronide
30	12.8	269, 332	537	493 (10) [M-H-CO_2]$^-$<br>375 (100) [M-H-$C_9H_6O_3$]$^-$<br>331 (6) [M-H-$C_{10}H_6O_5$]$^-$	Biapigenin (Amentoflavone)
31	13.4	269, 351	491	476 (9) [M-H-CH_3]$^-$<br>329 (100) [tricin-H]$^-$	Tricin-7-*O*-hexoside
32	13.5	271, 339	431	341 (28) [M-H-90]$^-$<br>311 (100) [M-H-120]$^-$	Apigenin-6-*C*-hexoside
33	13.9	270, 350	461	371 (26) [M-H-90]$^-$<br>341 (100) [M-H-120]$^-$<br>MS^3[341]:<br>313 (100) [M-H-CO]$^-$<br>299 (49) [Trihydroxymethoxy flavone-H]$^-$	Trihydroxymethoxy flavone *C*-hexoside (isomer IV)
34	14.1	272, 332	687	525 (100) [M-H-hexose]$^-$<br>329 (8) [tricin-H]$^-$	Tricin guaiacylglyceryl hexoside (isomer I)

35	14.3	271, 335	431	341 (10) [M-H-90]-<br>311 (100) [M-H-120]-	Apigenin-8-*C*-hexoside
36	14.6	272, 338	687	525 (100) [M-H-hexose]$^-$<br>329 (8) [tricin-H]$^-$	Tricin guaiacylglyceryl hexoside (isomer II)
37	14.7	271, 354	461	371 (8) [M-H-90]$^-$<br>341 (100) [M-H-120]$^-$<br>MS^3[341]:<br>313 (100)[M-H-CO]$^-$<br>299 (62) [Trihydroxymethoxy flavone-H]$^-$	Trihydroxymethoxy flavone *C*-hexoside (isomer V)
38	15.0	242, 278	373	329 (100) [tricin-H]$^-$	3-*O*-Acetyl tricin
39	15.1	283, 327	611	593 (18) [M-H-H_2O]$^-$<br>449 (100) [M-H-hexose]$^-$<br>MS^3[449]:<br>287 (100) [eriodictyol-H]$^-$	Erioctioyl di-*O*-hexoside
40	15.2	248, 351	409	329 (100) [tricin-H]$^-$	Tricin sulfate
41	15.6	276, 340	629	611 (6) [M-H-H_2O]$^-$<br>467 (100) [M-H-hexose]$^-$<br>MS^3[467]:<br>313 (100) [Trihydroxymethylenedioxyflavone-H]$^-$	Trihydroxymethylenedioxyflavone dihydrogalloyl hexoside
42	16.0	271, 336	605	329 (100) [tricin-H]$^-$<br>314 (10) [tricin-H-CH_3]$^-$	Tricin guaiacylglycerylsulfate
43	16.4	271	403	388 (9) [M-H-CH_3]$^-$<br>329 (100) [tricin-H]$^-$	Tricin glyceryl
44	16.5	274, 368	507	492 (100) [M-H-CH_3]$^-$<br>339 (12) [Hydroxydimethoxydimethyl homoisoflavone -H]$^-$<br>311 (8) [Hydroxydimethoxydimethyl homoisoflavone -H-CO]$^-$	Hydroxydimethoxydimethyl homoisoflavone-C-methylgalloyl
45	16.7	277, 368	691	497 (100) [M-H-glucuronic acid]$^-$<br>329 (60) [tricin-H]$^-$<br>314 (10) [tricin-H-CH_3]$^-$	Tricin *C*-methylgalloyl glucuronide
46	16.8	279, 338	417	373 (10) [M-H-CO_2]$^-$<br>354 (21)<br>329 (100) [tricin-H]$^-$	Tricin derivative
47	16.9	279, 320	643	481 (100) [M-H-hexose]$^-$<br>329 (20) [M-H-hexose-galloyl]$^-$<br>314 (12) [tricin-H-CH_3]$^-$<br>299 (10) [tricin-H-2CH_3]$^-$	Tricin-*C*-galloyl-*O*-hexoside
48	17.6	269, 347	329	314 (100) [tricin-H-CH_3]$^-$<br>299 (7) [tricin-H-2CH_3]$^-$	Tricin
49	17.9	248, 339	588	571(87) [M-OH]$^-$<br>439 (100) [quercetagetin trimethyl *O*-sulfate -H]$^-$<br>359 (16) [quercetagetin trimethyl-H]$^-$	Quercetagetin trimethyl *O*-sulfate pentoside
50	18.0	271, 336	525	329 (100) [tricin-H]$^-$	Tricin-4-*O*-guaiacylglyceryl
51	18.2	271, 366	541	495 (100) [M-H-CO-H_2O]$^-$<br>345 (19) [spinacetin-H]$^-$	Spinacetin guaiacylglyceryl
52	19.1	275, 362	569	551 (34) [M-H-H_2O]$^-$<br>345 (100) [spinacetin-H]$^-$	Spinacetin sinapoyl
53	19.9	271, 332	853	805 (12) [M-H-CH_2O-H_2O]$^-$<br>493 (100) [M-H- secoisolariciresinol]$^-$<br>329 (54) [tricin-H]$^-$<br>314 (13) [tricin-H-CH_3]$^-$<br>299 (12) [tricin-H-2CH_3]$^-$	Tricin secoisolariciresinol coumaroyl
54	20.1	280	817	577 (60) [procyanidin dimer-H]$^-$<br>559 (88)[procyanidin dimer-H-H_2O]$^-$<br>537 (42)<br>451 (46)<br>407 (100)	Procyanidyn dimer derivative
55	20.4	262, 362	599	447 (100) [M-H-galloyl]$^-$<br>285 (47) [kaempferol-H]$^-$	Kaempferol galloyl hexoside
56	20.5	262, 332	599	584 (100) [M-H-CH_3]$^-$<br>419 (16) [M-H-caffeoyl]<br>283 (18) [methylgnistein-H]$^-$	Methylgnistein caffeoyl derivative
57	21.0	271, 320	641	623 (100) [M-H-H_2O]$^-$<br>445 (32) [M-H-guaiacylglyceryl]$^-$	Dihydroxyflavanone Acetyl guaiacylglyceryl galloyl

				293 (30) [acetyl dihydroxyflavanone-H]$^-$<br>255 (21) [dihydroxyflavanone-H]$^-$	
58	21.7	254, 265	577	415 (22) [M-H-hexose]$^-$<br>299 (100) [trihydroxymethoxyflavone-H]$^-$	Trihydroxymethoxyflavone-*O*-hexose-*O*-malloyl
59	22.1	257, 272	649	603 (10) [M-H-CO-H_2O]$^-$<br>487 (51) [M-H-hexose]$^-$<br>413 (82) [M-H-hexose-glycerol]$^-$<br>299 (100) [trihydroxymethoxy flavone-H]$^-$	Trihydroxymethoxy flavone *O*-glycosylhexoside-*O*-5-hydroxy-4-mehoxypentanoic acid
60	22.4	260, 371	540	480 (100) [M-H-acetate]$^-$<br>304 (9) [methyl catechin -H]$^-$	*O*-Methylcatechin acetate glucuronide
			Others		
61	2.8	274	241	197 (100) [syringic acid-H]$^-$	Syringic acid derivative
62	9.5	216, 278	535	197 (21) [syringic acid-H]$^-$<br>163 (8) [coumaric acid-H]$^-$<br>129 (10)<br>85 (100)	Coumaroylsyringylglucarate acid
63	12.9	271, 339	683	521 (42) [M-H-hexose]$^-$<br>359(27) [lariciresinol-H]$^-$<br>329 (100) [lariciresinol-H-2CH_3]$^-$	Lariciresinol dihexoside
64	14.8	223	231	213 (100) [M-H-H_2O]$^-$<br>187(10) [M-H-CO_2]$^-$	Costunolide
65	18.4	250, 298	571	525 (100) [M-H-CO-H_2O]$^-$<br>MS^3[525]<br>437 (100) [dehydrated oleanolic acid-H]$^-$	Dehydrated oleanolic acid pentoside

Table S4. Linearity (y = mx + b, where y corresponds to the standard peak area and x corresponds to the mass of standard), LOD and LOQ of pure compounds used as reference

Standard compound	Range concentration	Slope (m)	Intercept (b)	R^2	LOD§	LOQ§
Benzoic acid	0.5-500	16748	111	0.9998	12	40
Gallic acid	0.5-500	557	-728	0.9988	11	37
Catechin	0.5-250	142	-58	0.9997	8	27
Caffeic acid	0.5-550	17	-411992	0.9992	10	34
p-Coumaric acid	0.5-550	46	-532140	0.9952	15	50
Ferulic acid	0.5-500	1633	6	0.9993	10	33
Chlorogenic acid	0.5-250	659	-8	0.9989	9	30
Rutin	0.5-100	26	10080	0.9986	3	10
Kaempferol	0.5-175	792	-76	0.9969	5	17
Luteolin	0.5-150	354	-221	1.0000	3	10
Quercetin	0.5-200	46	-390882	0.9989	4	13
Ursolic acid	0.5-250	167484	111	0.9995	15	50

§The detection and quantification limits (LOD and LOQ, respectively) were determined from the parameters of the calibration curves (LOD = 3 standard deviation/slope and LOQ = 10 standard deviation/slope), in μg/mL

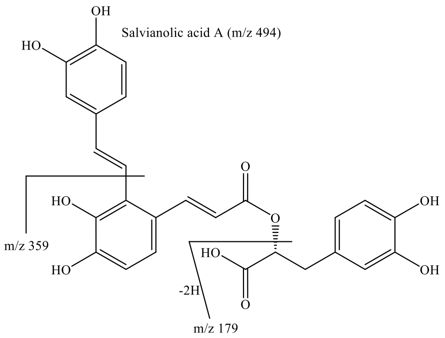

Fig. S3. Structure of salvianolic acid A (**8**) and its main fragments.

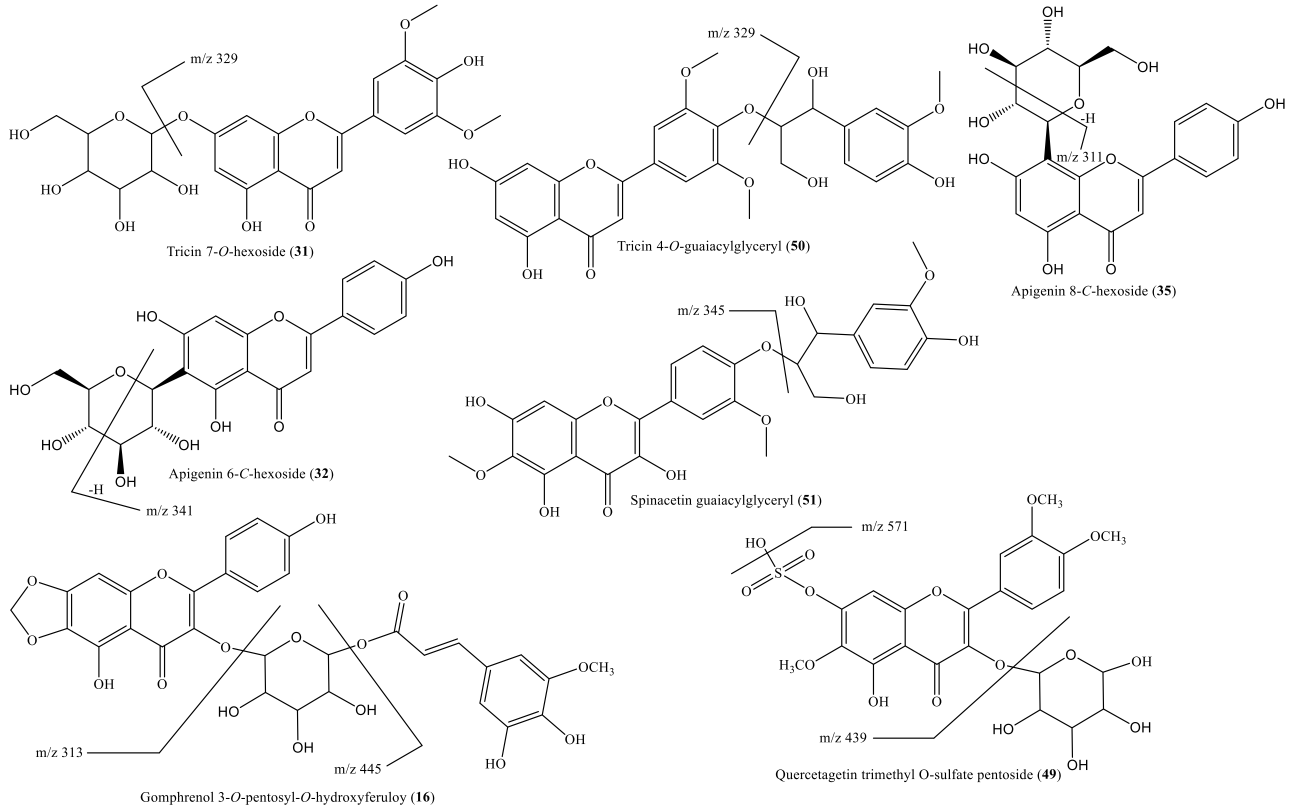

Fig. S4. Structure of some flavonoids identified in the polyphenolic extracts of the three studied *taxa*.

7. Extraction yield of the polyphenolic extracts

The yield of the ethanol extracts of *P. maritima, S. maritima,* and *S. patens* was 25.2%, 15.3% and 22.4% of dry plant, respectively. The differences in the yields, even between the same genus, are the first indication that the three *taxa* produce different quantities of ethanol soluble compounds. Interestingly, these values revealed to be more similar between *P. maritima* and *S. patens* than between the two *Spartina* species. Since previously reported extraction yields in these *taxa* are not available in the literature, a comparison of the results is not conceivable.

8. Total chlorophyll quantification

The concentration of chlorophyll *a* and *b* in the extracts was determined spectrophotometrically by placing the extract dissolved in methanol pa. in a quartz cuvette and measuring the absorbance spectra between 350 and 800 nm, in the UV-visible spectrometer (spectrometer Shimadzu UV-2501 PC,). The quantification of chlorophylls was achieved following Lichtenthaler equations[4]: $\mathbf{Ch_a}=16.77A_{665.2}-9.16A_{652.4}$; $\mathbf{Ch_b}=34.09A_{652.4}-15.28A_{665.2}$; $\mathbf{Ch_a}+\mathbf{Ch_b}=1.44A_{665.2}+24.93A_{652.4}$, being $\mathbf{Ch_a}$=chlorophyll *a*, $\mathbf{Ch_b}$=chlorophyll *b*, $A_{652.4}$=absorbance at 652.4 nm and $A_{665.2}$=absorbance at 665.2 nm.

In order to identify the best method for chlorophyll extraction, the total chlorophyll content of each extract was assessed base on spectrophotometric measurements. This technique is widely applied for this purpose[5,6] and is based on chlorophyll absorption bands at $\lambda < 460$ nm and in the range 630-670 nm. Spectroscopic technology provides fast, convenient and non-destructive detection.[7] Nonetheless, this quantification is not completely accurate since, degradation products, such as chlorophyllides and phaeopigments, have spectroscopic characteristics close to those of their parent chlorophylls.[8] The basic structure of chlorophylls is a tetrapyrrole macrocycle chelating a magnesium ion with a side phytol chain, in the case of chlorophylls *a* and *b* (present in higher plants).[9] Nonetheless, these molecules are heat labile and can degrade to form pheophytins and pyropheophytins.[10] In the first case, this degradation product is form from the displacement of the central magnesium atom from the chlorophyll porphyrin ring with hydrogen.[10,11] Pyropheophytins are formed from pheophytins after the loss of the carbomethoxy group at the C-10 position and are produced as a result of more extreme forms of heat treatment.[10,12]

As it is shown in **table S5** and **figure S5**, in both *S. patens* and *P. maritima* the extract with the highest concentration of chlorophylls was the one performed with microwave (0.21 mg/100 mg of extract and 0.65 mg/100 mg of extract, respectively). Contrarily, in *S. maritima* the room temperature stirring extract showed the higher chlorophyll concentration with 1.15 mg/100 mg of extract. These results are correlated to the ones of extraction yield (R=0.675), in which the higher extraction yields corresponded to the ones with higher chlorophyll concentration. Regarding the chlorophyll *a* and *b* content, *P. maritima* and *S. maritima* presented in higher quantities chlorophyll *a* while *S. patens* chlorophyll *b*. Chlorophyll *a* is essential in photosynthesis and chlorophyll *b* is an accessory pigment acting indirectly in photosynthesis by transferring the light it absorbs to chlorophyll *a*.[13] The differences herein observed regarding the content of chlorophylls are not surprising since, it is known that the content of these pigments vary greatly among species and are also related to internal and environmental factor.[9] Additionally, through a Tukey's test, the same statistical significant differences as in **EY** were obtained. In the case of *P. maritima* and *S. patens*, statistically significant differences were observed only between microwave and ultrasound ($p<0.05$). *S. maritima*'s extracts only showed significant differences between room temperature stirring and ultrasound extractions ($p<0.05$).

Furthermore, a relationship between total chlorophyll content and salinity stress has been achieved. It has been reported that increased concentrations of NaCl on the external medium result in decreased chlorophyll content.[14-17] It appears that reduced photosynthesis and the subsequent decreased growth under stress

conditions, generally result from the reduction in chlorophyll content.[18] By comparison with other halophytic grasses, it seems that microwave assisted extraction improves chlorophyll extraction. For instance, the chlorophyll content on *S. maritima* (acetone extract) was 0.4 mg/ g fresh weight,[19] in our case these values were of 0.51 mg/g fresh weight in room temperature stirring technique and 0.23 in microwave assisted extraction for the same plant. Additionally, in these species, the values of chlorophylls do not seem to change significantly in salinity stress.[20]

Table S5. Chlorophyll *a* (**Ch_a**), *b* (**Ch_b**) and total (**Ch_{a+b}**) content (mg/100 mg of extract) of *P. maritima*'s ethanol extracts from microwave (**PMMW**), room temperature stirring (**PMST**) and ultrasound (**PMUS**) as well as *S. maritima* microwave (**SMMW**), room temperature stirring (**SMST**) and ultrasound (**SMUS**) extracts and *S. patens* microwave (**SPMW**), room temperature stirring (**SPST**) and ultrasound (**SPUS**) extracts.

	PMMW	PMST	PMUS	SMMW	SMST	SMUS	SPMW	SPST	SPUS
Ch_a	0.15	0.07	0.05	0.37	0.82	0.27	0.02	0.01	0.03
Ch_b	0.06	0.06	0.04	0.15	0.29	0.13	0.63	0.60	0.45
Ch_{a+b}	0.21	0.13	0.09	0.53	1.15	0.40	0.65	0.61	0.48

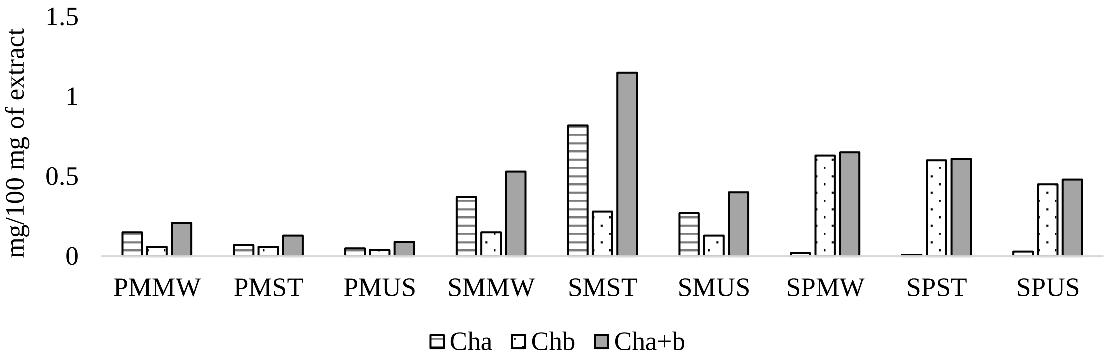

Figure S5. Chlorophyll *a* (**Ch_a**), *b* (**Ch_b**) and total (**Ch_{a+b}**) content (mg/100 mg of extract) of *P. maritima*'s ethanol extracts from microwave (**PMMW**), room temperature stirring (**PMST**) and ultrasound (**PMUS**) as well as *S. maritima* microwave (**SMMW**), room temperature stirring (**SMST**) and ultrasound (**SMUS**) extracts and *S. patens* microwave (**SPMW**), room temperature stirring (**SPST**) and ultrasound (**SPUS**) extracts.

9. Extraction yield of the chlorophyll rich extracts

The extraction yields (mass of extract/mass of dry matter x 100) are considered indicators of the effects of the extraction conditions and proceeding,[21] therefore the comparison of their values among different extracts can be indicative of the most suitable extraction procedure applied. The yields of the ethanol extracts achieved by microwave, ultrasound, and room temperature stirring techniques of *S. maritima*, *P. maritima* and *S. patens* are summarized in **table S6** and **figure S6**. In both *S. patens* and *P. maritima* (**Figure S6** and **Table**

S6), the highest extraction yield (**EY**) was observed in microwave assisted extraction (2.3% and 2.5%, respectively). Contrarily, *S. maritima* showed the best **EY** with room temperature stirring technique, although this value (4.5%) is similar to the one obtained by microwave-assisted extraction (4.1%). Ultrasound assisted extraction showed the lowest **EY** in all studied *taxa*. This is the first sign that the microwave technique seems to improve the chlorophyll extraction. Even in the case of *S. maritima*, the **EY** is closest to the highest one observed. Taking into consideration the time reduction involved in extraction, since microwave was performed during 10 min and room temperature stirring for 48 h, this could drastically improve the process.

Table S6. Extraction yield (%) of *P. maritima*'s ethanol extracts from microwave (**PMMW**), room temperature stirring (**PMST**) and ultrasound (**PMUS**) as well as *S. maritima* microwave (**SMMW**), room temperature stirring (**SMST**) and ultrasound (**SMUS**) extracts and *S. patens* microwave (**SPMW**), room temperature stirring (**SPST**) and ultrasound (**SPUS**) extracts.

	PMMW	PMST	PMUS	SMMW	SMST	SMUS	SPMW	SPST	SPUS
Extraction yield (%)	2.34 ± 0.02	1.45 ± 0.01	0.35 ± 0.01	4.11 ± 0.03	4.47 ± 0.04	0.97 ± 0.01	2.50 ± 0.02	2.00 ± 0.01	0.64 ± 0.01

In order to confirm the differences among the **EY** obtained with the different techniques, a Tukey's test was performed. In the case of *P. maritima* and *S. patens*, statistically significant differences were observed only between microwave and ultrasound ($p<0.05$). *S. maritima*'s extracts only showed significant differences between room temperature stirring and ultrasound extractions ($p<0.05$). Additionally, it is evident that *S. maritima* presents more quantity of ethanol soluble pigments compared to the other species, since its extraction yields, in all techniques applied, are considerably higher.

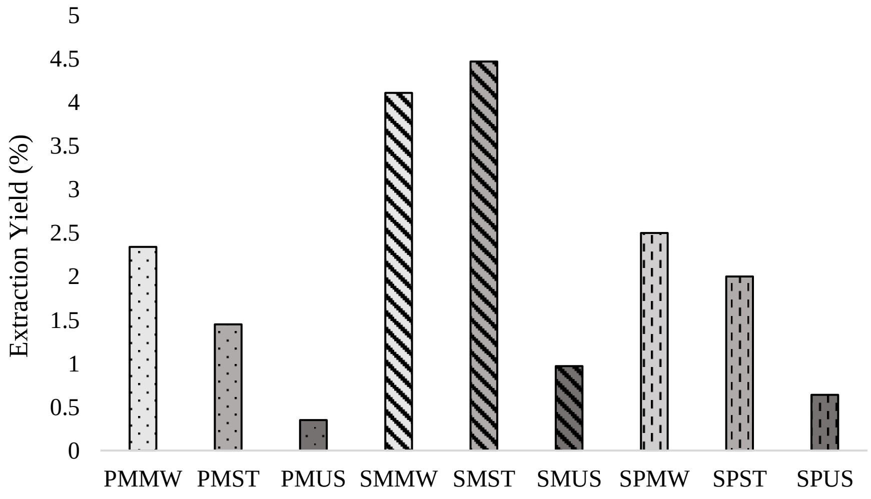

Figure S6. Extraction yield (%) of *P. maritima*'s ethanol extracts from microwave (**PMMW**), room temperature stirring (**PMST**) and ultrasound (**PMUS**) as well as *S. maritima* microwave (**SMMW**), room temperature stirring (**SMST**) and ultrasound (**SMUS**) extracts and *S. patens* microwave (**SPMW**), room temperature stirring (**SPST**) and ultrasound (**SPUS**) extracts.

These results are in agreement with the literature data in which is stated that microwave assisted extraction presents several advantages over traditional techniques. Among these, lower energy consumption, less production of waste and higher extraction yields are included.[22] The improved **EY** are associated with protein denaturation of the cell during extraction, which ultimately results in higher yields.[23] Furthermore, during microwave assisted extraction, the disruption of the cells occur and a rapid exudation of chemical substances from cells intro surrounding extractant is observed.[24] Several studies reported a decrease in extraction time and increase in extraction yield using this method.[25,26]

Contrarily to the results herein obtained, ultrasound assisted extraction is also associated with better **EY**. In this extraction, the combination of pressure, heat, turbulence, and mechanical mixing, caused by ultrasonic waves, are used to accelerate mass transfer in the extraction process, reducing extraction time an increasing extraction yield.[27,28] In addition, thermal decomposition of heat-sensitive compounds is avoided in ultrasound since it is a non-thermal process.[27] The low extraction yield obtained with this method could be mainly related to two factor[29]: (1) temperature, it has been reported that increase in this variable correlates with improvement in **EY** due to induction of matrix bonds ruptures, increase of the compound solubility, solvent diffusion rate and mass transfer.[29,30] The temperature reported associated with good **EY** are between 40 and 80 ºC and the temperature herein used was 30 ºC;[30,31] (2) extraction time, since, the total content extracted by ultrasound increases as a function of time. In the first 10-20 min, 90% of the total extractable compounds can be achieved.[32] Notwithstanding, the process employed by us was only performed for 10 min. Therefore, these factors might have influenced the procedure.

Table S7. Antioxidant capacity and acetylcholinesterase inhibitory activity of *P. maritima*, *S. maritima*, and *S. patens* ethanol extracts.

Species	Total phenolic content (UHPLC-MS) mg/100mg of extract	**DPPH**$^{•}$ (IC_{50}) (µg/mL)	**ABTS**$^{+•}$ (IC_{50}) (µg/mL)	Reducing power (IC_{50}) (µg/mL)	% inhibition of acetylcholinesterase (100 µg/mL)
P. maritima	13.13 ± 0.28	373.45 ± 28.63$^{*,\#}$	81.09 ± 6.24$^{**,\#}$	> 500	4.17 ± 0.00
S. maritima	23.12 ± 0.78	317.46 ± 35.68$^{*,\#}$	67.56 ± 12.82$^{*,\#}$	> 500	5.83 ± 0.28$^{\#\#\#}$
S. patens	15.77 ± 0.22	207.63 ± 10.50$^{*,\#\#,\#\#\#}$	37.13 ± 1.44$^{**,\#\#,\#\#\#}$	> 500	5.71 ± 0.27$^{\#\#\#}$
Reference compound IC_{50}		6.37 ± 0.19 (Trolox)	2.98 ± 0.11 (Trolox)	17.22 ± 0.31 (Trolox)	0.016 ± 0.01 µg/mL (Donepezil)

*Statistically significant different with respect to standard compound (Tukey's test), p<0.001
** Statistically significant different with respect to standard compound (Tukey's test), p=0.002
$^{\#}$ Statistically significant different with respect to *S. patens* (Tukey's test), p<0.001
$^{\#\#}$ Statistically significant different with respect to *S. maritima* (Tukey's test), p<0.001
$^{\#\#\#}$ Statistically significant different with respect to *P. maritima* (Tukey's test), p<0.001

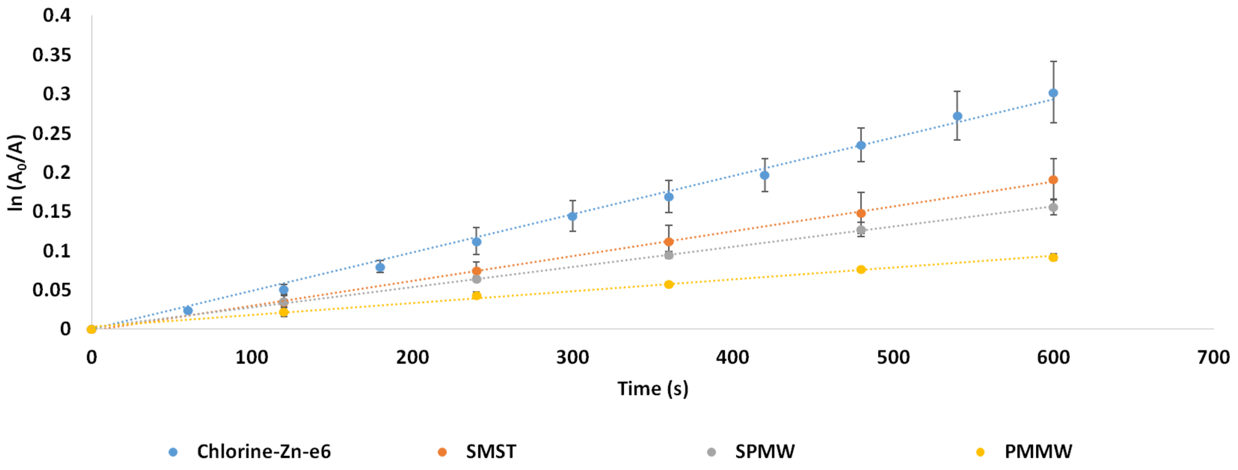

Figure S7. Graphical representation of ln (A_0/A) in function of time (s) of positive control (Znchlorin e_6, negative control, and of the extracts **SMST**, **SPMW** and **PMMW**.

Table S8. Percentage of photodegradation in **PMMW**, **SMST** and **SPMW** after incidence for 5, 15, 35 and 75 min with red light (630 ± 20 nm, 10 mW cm^{-2}) and for 15 and 30 min with white light (400-800 nm, 50 mW cm^{-2}).

Time (min)	**PMMW**	**SMST**	**SPMW**
		**Red light (10 mW cm^{-2})**	
5	0%	6%	7%
15	0%	12%	11%
35	0%	19%	14%
75	0%	25%	18%
		**White light (50 mW cm^{-2})**	
15	9%	17%	7%
30	13%	30%	11%

References

1 M. V. Faustino, D. C. G. A. Pinto, M. J. Gonçalves, L. Salgueiro, P. Silveira and A. M. S. Silva, *J. Func. Foods*, 2018, **45**, 254-267.

2 M. V. Faustino, A. M. L. Seca, P. Silveira, A. M. S. Silva and D. C. G. A. Pinto). *Ind. Crops Prod.*, 2017, **104**, 91-98.

3 A. Wojdyło, J. Oszmiański and R. Czemerys, *Food Chem.*, 2007, **105**, 940-949.

4 H. K. Lichtenthaler, Method. Enzymol., 1987, **148**, 352-382.

5 A. Picazo, C. Rochera, E. Vincente, M. R. Miracle and A. Camacho, *Limnetica*, 2013, **32**, 139-158.

6 J.-E. Thrane, M. Kyle, M. Striebel, S. Haande, M. Grung, T. Rohrlack and T. Andersen, *PLoS ONE*, 2015, **10**, e0137645.

7 B. Liu, Y.-M. Yue, R. Li, W.-J. Shen and K.-L. Wang, *Sensors*, 2014, **14**, 19910-19925.

8 A. A. Gitelson and M. N. Merzlyak, *Remote Sens. Agr. Environm.*, 2004, **2004**, 78-94.

9 A. Bannari, K. S. Khurshid and K. Staenz, *Geosic. Remote Sens.*, 2007, **45**, 3063-3073.

10 G. Pumilia, M. J. Cichon, J. L. Cooperstone, D. Giuffrida, G. Dugo and S. J. Schwartz, *Food Res. Internat.*, 2014, **65**, 193-198.
11 M. Bellomo and B. Fallico, *J. Food Compos. Anal.*, 2007, **20**, 352-359.
12 F. L. Canjura, S. J. Schwartz and R. V. Nunes, *J. Food Sci.*, 1991, **56**, 1639-1643.
13 M. A. Costache, G. Campeanu and G. Neata, *Romanian Biotecnolo. Lett.*, 2012, **17**, 7702-7708.
14 Y. Ali, Z. Aslam, M. Y. Ashraf and G. R. Tahir, *Inter. J. Environm. Sci. Technol.*, 2004, **1** 221-225.
15 M. Heidari, *African J. Biotechnol.*, 2011, **11**, 379-384.
16 M. Rabhi, A. Castagna, D. Remorini, C. Scattino, A. Smaoui, A. Ranieri and C. Abdelly, *South African J. Bot.*, 2012, **79**, 39-47.
17 K. Taïbi, F. Taïbi, L. A. Abderrahim, A. Ennajah, M. Belkhodja and J. M. Mulet, *South Afric. J. Bot.*, 2016, **105**, 306-312.
18 H. Mohammadi and J. Kardan, *Pobrane z Czasopisma Annales*, 2015, **2**, 31-41.
19 B. Duarte, T. Couto, J. Freitas, J. Valentim, H. Silva, J. C. Marques and I. Caçador, *Estuar., Coast. Shelf Sci.*, 2013, **130**, 127-137.
20 J. Ma, M. Chai and F. Si, *Afric. J. Biothecnol.*, 2011, **10**, 17962-17968.
21 T. Dhanani, S. Shah, N. A. Gajbhiye and S. Kumar, S. *Arabian J. Chem.*, 2017, **10**, S1193-S1199.
22 E. C. Creencia, J. A. P. Nillama and I. L. Librando, *Resources*, 2018, **7**, 1-12.
23 P. Tatke and Y. Jaiswal, *Res. J. Med. Plants*, 2011, **5**, 21-31.
24 H.-F. Zhang, X.-H. Yang and Y. Wang, *Trends Food Sci. Technol.*, 2011, **22**, 672-688.
25 M. Gallo, R. Ferracane, G. Graziani, A. Ritieni and V. Fogliano, *Molecules*, 2010, **15**, 6365-6374.
26 S. Tsubaki, M. Sakamoto and J. Azuma, *Food Chem.*, 2010, **123**, 1255-1258.
27 A. E. Ince, S. Sahin and G. Sumnu, G. *J. Food Sci. Technol.*, 2014, **51**, 2776-2782.
28 R. Vardanega, D. T. Santos and M. A. A. Meireles, *Pharmacogn. Rev.*, 2014, **8**, 88-95.
29 N. Medina-Torres, T. Ayora-Talavera, H. Espinho-Andrews, A. Sánchez-Contreras and N. Pacheco, *Agronomy*, 2017, **7**, 1-19.
30 G. B. Celli, A. Ghanem and M. S.-L. Brooks, *Ultrason. Sonochem.*, 2015, **27**, 449-455.
31 M. B. Hossain, N. P. Brunton, A. Patras, B. Tiwari, C. P. O'Donnell, A. B. Martin-Diana and C. Barry-Ryan, *Ultrason. Sonochem.*, 2012, **19**, 582-590.
32 S. Şahin and R. Samlı, *Ultrason. Sonochem.*, 2013, **20**, 595-602.
